# Cohesive versus Flexible Evolution of Functional Modules in Eukaryotes

**DOI:** 10.1371/journal.pcbi.1000276

**Published:** 2009-01-30

**Authors:** Like Fokkens, Berend Snel

**Affiliations:** 1 Theoretical Biology and Bioinformatics, Utrecht University, Utrecht, The Netherlands; 2 Academic Biomedical Centre, Utrecht University, Utrecht, The Netherlands; University of Zurich, Switzerland

## Abstract

Although functionally related proteins can be reliably predicted from phylogenetic profiles, many functional modules do not seem to evolve cohesively according to case studies and systematic analyses in prokaryotes. In this study we quantify the extent of evolutionary cohesiveness of functional modules in eukaryotes and probe the biological and methodological factors influencing our estimates. We have collected various datasets of protein complexes and pathways in *Saccheromyces cerevisiae*. We define orthologous groups on 34 eukaryotic genomes and measure the extent of cohesive evolution of sets of orthologous groups of which members constitute a known complex or pathway. Within this framework it appears that most functional modules evolve flexibly rather than cohesively. Even after correcting for uncertain module definitions and potentially problematic orthologous groups, only 46% of pathways and complexes evolve more cohesively than random modules. This flexibility seems partly coupled to the nature of the functional module because biochemical pathways are generally more cohesively evolving than complexes.

## Author Summary

Components of a protein complex or a metabolic pathway strongly cooperate to perform a specific function. Because of this functional interdependence, proteins that form a complex or pathway are expected to be present and absent together in different species. Phylogenetic profiling methods, in which proteins with similar presence and absence patterns are inferred to be functionally linked, are based on this assumption. In this report, we quantify to what extent proteins that together constitute a complex or pathway (a functional module) in yeast are present and absent together (evolve cohesively) in other eukaryotic species. We find that more than half of all complexes and pathways are only partially present in a number of species. It appears that evolution of functional modules is very flexible; components are not indispensable; they can be replaced or reused in a different functional context. This places a limit on how well phylogenetic profiling methods can detect functionally related proteins. Functional modules that evolve cohesively are typically involved in biological processes such as translation and amino acid metabolism.

## Introduction

Phylogenetic profiling is a successful method to predict or confirm functional relations between proteins. If the phylogenetic patterns of two proteins are alike, they are likely to be functionally related [1]. However, this does not necessarily mean that all functionally related proteins have similar phylogenetic patterns. In depth phylogenetic reconstructions of specific pathways and complexes have yielded a number of examples of complexes and pathways gradually gaining and losing components during evolution [2]–[8]. A preponderance of flexible evolution has also been suggested by a number of large scale studies in prokaryotes [9]–[11]. Both types of studies thus reveal limited modularity or ‘cohesiveness’ in evolution of functional modules, showing that the flexibly evolving examples are not an exception.

Recent application of phylogenetic profiling methods on eukaryotes has not been as successful in identifying functional relations as in prokaryotes [12]. This raises the question to what extent, if at all, functional modules evolve cohesively in eukaryotes. The organization of bacterial genomes into operons should facilitate modular evolution of functionally linked proteins. In eukaryotes however, gene order and genome organization are unlikely to play an important role and any modular coevolution would be the result of nongenomic, e.g. system level, properties of the functional module. The study of evolutionary cohesiveness of functional modules in eukaryotes may therefore enable us to shed new light on the way functional organization influences the evolutionary dynamics of the genome and vice versa. The recent availability of a sufficient number of sequenced and assembled genomes across the eukaryotic species tree, as well as the accessibility of high throughput functional data, yield the opportunity to look at possible cohesive evolution in eukaryotes in a systems biological context.

Our aims in this study are twofold: we want to define and quantify evolutionary cohesiveness of functional modules in eukaryotes, and, given this quantification, we want to understand the evolutionary behavior which we observe. In order to meet these goals, we collect a diverse set of functional modules (pathways and complexes). For each module we describe the evolutionary dynamics of its constituents across 34 species from 6 major eukaryotic divisions. We select a measure to determine from the dynamics whether we should consider a module to display cohesive evolution. Once this quantification of the degree of cohesive evolution of functional modules in eukaryotes is established, we are able to compare cohesively with flexibly evolving modules and gain insight in both methodological as well as biological factors which contribute to our result.

## Results

### Scoring Cohesiveness

**Figure 1:**
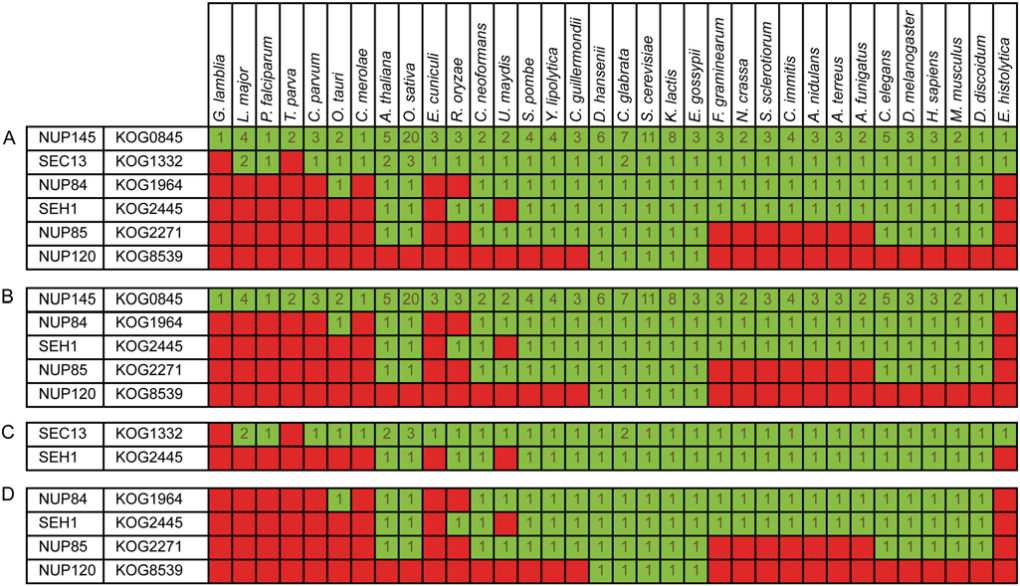
Example of a flexibly evolving complex: Nup84 subcomplex of the nuclear pore complex. (A) The profile of the Nup84 complex, red indicating absence, green presence (number of paralogs in dark green). The raw score of this complex is (5,0), which means that there are 5 species in which this complex is completely present and none in which this complex is completely absent. The cohesiveness score, which is the fraction of random modules of the same size which score better both in the number of species in which the module is present as well as in the number of species in which the module is absent, is 0.48. This complex from the Aloy dataset occurs also in the MIPS dataset and, with some additional subunits, in the PE and Socio-affinity clusters, so it passes the cross-comparison filter without losing any subunits. (B) The profile after cross-comparison with TAP data. SEC13, which is also part of the COPII complex, has the lowest PE score with the other subunits and has a higher propensity to interact with a protein outside the module (namely with SEC31, an other member of the COPII complex) than with any other member of this module. Removal of this protein from the module results in a subcomplex which is not evolving more cohesively than the original module. (C) Apart from improving the module definition, we attempt to filter possible noise originating from the use of orthologous groups to describe a modules evolutionary dynamics. KOG0845, KOG1964, KOG2271 and KOG8539 are considered unreliable because they have less than 90% overlap with a orthoMCL derived orthologous group. Removal of those orthologous groups leads to a more cohesively evolving module, with a raw score (24,2) and a cohesiveness score 0.87. (D) Removal of orthologous groups which are likely to have functionally differentiated (groups containing many inparalogs, in this example KOG0845 and KOG1332) results in a submodule which we consider evolutionary cohesive: it has a raw score of (5,8) and a cohesiveness score of 0.996. More details on this module and some additional examples can be found in Text S2.

We gather 6 datasets containing protein complexes and pathways, defined in *S. cerevisiae*, as our set of functional modules (Table 1). In order to measure coevolution of the components of a functional module, we assign all proteins which are part of a module to orthologous groups, based on predefined euKaryotic Orthologous Groups (KOGs) [13], for all proteins from 34 eukaryotic species (see Materials and Methods), resulting in 214.342 (out of 368.358, >58%) assigned proteins. The (partial) presence or absence of a module in a species depends on whether there are proteins from that species assigned to the orthologous group to which the module components belong (Figure 1).

**Table 1:** Datasets used in this study.

Dataset	Number of Modules	Average Module Size
SGD	106	4.56
KEGG	92	14.89
MIPS	199	5.91
Aloy	87	6.95
PE	433	4.37
Socio-affinity	461	11.15
All	1285	8.02
All curated	447	7.51

The number of modules and the average number of subunits in the modules are listed per dataset, as well as for the nonredundant combination of all datasets (‘all’) and of all curated datasets (‘all curated’). The SGD pathways and KEGG datasets are curated and consist mainly of metabolic pathways. The PE and socio-affinity datasets both result from clustering Tandem Affinity Purification (TAP) data. The differences between these two datasets include the fact that PE clusters are based on raw data from the study by Krogan et al. [30] as well as from Gavin et al. [14], the similarity score (Purification Enrichment versus Socio-affinity) and the algorithm used to cluster the proteins. MIPS and Aloy are two curated complex datasets, the Aloy dataset is a manual selection based on extensive literature curation, information on protein structures and previous TAP derived protein complexes [26]. Curated datasets comprise approximately one third of all modules.

No standard method exists to measure the degree to which a module evolves cohesively. Hence we implement several scoring schemes, both from the literature as well as newly defined. We compare individual modules to a random background in order to decide whether a pattern is the result of evolutionary dynamics or could have been obtained randomly. We adopt the strategy from Campillos et al. [11]: for each size N of functional modules, we generate 100.000 random modules by randomly selecting N groups from the set of orthologous groups which are part of at least one functional module. Each functional module is assigned a cohesiveness score defined as the fraction of random modules with a lower ‘raw’ score. At a cutoff of 0.99, reflecting a probability to obtain a pattern this cohesive by chance of 0.01, we regard a functional module to evolve cohesively.

**Figure 2:**
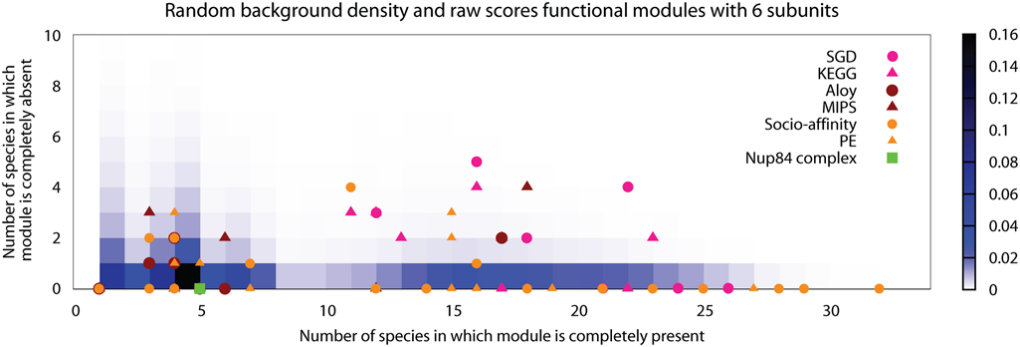
Scores and random background. This figure shows the raw scores for modules composed of six subunits from all datasets, with the Nup84 complex from Figure 1 highlighted in green. The random background density for all score bins is shown in shades of blue, turning darker as the number of random modules with a score in that particular bin increases.

We observe that regardless of the specific scoring scheme implemented, the majority of functional modules evolve flexibly (Table 2). In the remainder of our investigation we use the score which is most successful in separating real from random modules. This turns out to be a two dimensional vector consisting of the number of species in which the module is completely present and the number of species in which the module is completely absent (Figure 2). This score identifies 27% of all modules and 37% of all curated modules as cohesively evolving.

**Table 2:** Fraction of cohesive modules for different datasets and different scoring schemes.

Dataset	Average Cooccurrence	Average Deviation from Modular	Homogeneous Columns	Species Absent	Species Present	Species Absent, Species Present
SGD	0.14	0.15	0.09	0.06	0.03	0.44
KEGG	0.24	0.24	0.17	0.08	0.16	0.38
MIPS	0.17	0.17	0.15	0.05	0.1	0.33
Aloy	0.21	0.23	0.16	0.02	0.1	0.31
PE	0.08	0.08	0.06	0.03	0.05	0.21
Socio-affinity	0.27	0.3	0.2	0.01	0.19	0.24
All	0.18	0.2	0.14	0.03	0.12	0.27
All curated	0.19	0.19	0.15	0.06	0.1	0.37

Average Cooccurrence: for each pair of module subunits we calculate the fraction of species in which both subunits are either present or absent together. We average over all component pairs to obtain a score per module. Average deviation from modular: the sum of the deviation of the number of components of the functional module for each genome to the average number of module components per genome, adopted from Snel et al. [9]. Homogeneous Columns: the number of species in which a module is either completely present or completely absent, adopted from Gavin et al. [14]. Species Present, Species Absent: the number of species in which a module is completely present and the number of species in which the module is completely absent. Those two values together make up the raw score which is used throughout the article.

An additional merit of this score is that it does not correlate with module size, in contrast to other scores that seem to benefit larger modules (table 2 in Text S1). This is linked to a difference between cohesive large and small cohesive modules: manual inspection reveals that large modules typically distinguish themselves from the random background by being completely present in several species, while they're usually never completely absent. Yet small modules distinguish themselves from the random modules by being completely absent in at least a few species.

We carried out the quantification of cohesiveness in eukaryotes and, similarly to what has been observed previously in prokaryotes, we observe that the majority of functional modules evolves flexibly: 27% evolves cohesively on average, ranging from 21%–33% of complexes to 38%–44% of biochemical pathways. There is a host of potential technical and biological reasons for this observation. Are most of our pathways and complexes in fact not functional modules? Is functional modularity defined more appropriately on a different level (domain, protein, network)? Can proteins be functionally related but not co-evolving, because the intrinsic nature of their relationship makes it plastic in evolution? Does the time-span in our orthologous groups allow for so many duplications and subsequent independent losses that the real evolutionary history of the module is obscured?

The effects of these potential causes are difficult to disentangle. Nevertheless, we will attempt to assess the relative importance of module and orthologous group definition in the remainder of this study, in order to get a better estimate of the extent of cohesive evolution of complexes and pathways in eukaryotes. We improve our module definition by cross-comparison of our different datasets and by filtering our modules with data on interactions and cellular locations. Subsequently, we filter out those orthologous groups which are most likely to obfuscate the evolutionary history of a module component. Finally, we will discuss differences in characteristics between cohesively and flexibly evolving modules in order to gain further insights into the why of this observed level of flexibility.

### Effects of Module Definition

The fractions of cohesive modules per dataset as listed in Table 2 reveal a considerable disparity in the degree of evolutionary cohesiveness among datasets when they are different with respect to their underlying concepts. In contrast, results on datasets of the same category (‘pathways’, ‘curated complexes’ or ‘complexes based on high throughput data’) are much more congruent. These results suggest that curated datasets are of better quality compared to high throughput data based module definitions. Hence part of the flexible evolution observed here could be just a matter of poor module definition, as has been suggested previously [9]. We explicitly test this by applying different filters to enhance our module definition and see whether the level of cohesiveness is increased.

**Figure 3:**
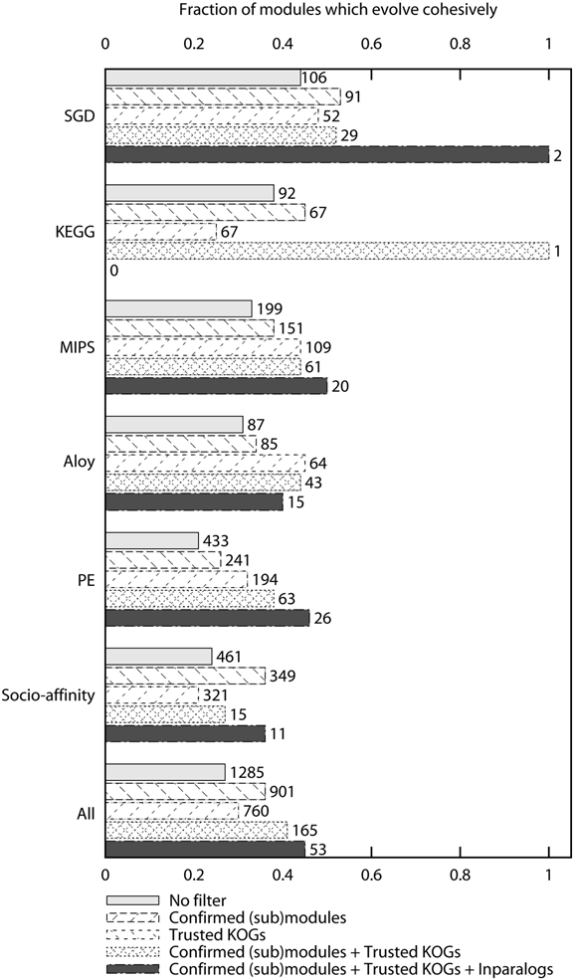
(Combined) effect of different filters on the fraction of cohesive modules. On top of each bar we show the number of (sub)modules passing the filter.

First we find that modules which are defined in multiple datasets tend to evolve more cohesively than modules which are not (P = 8e-06, table 4a in Text S1). Second we probed for a functional core that evolves more cohesively by trimming from all functional modules the parts that do not overlap with at least one other module definition. We observe that confirmed submodules evolve more cohesively than the original modules (P = 0.001, table 4b in Text S1), especially in those datasets containing large modules. Moreover the fraction of cohesive modules increases from 27% to 36% (Figure 3 and table 4c in Text S1). The primary observation that curated modules seem to evolve more cohesively than modules inferred from high throughput data, is bolstered by an increase in the extent of evolutionary cohesiveness after application of a cross-comparison filter. The combined evidence thus strongly suggests that part of the observed evolutionary flexibility can be attributed to the incorrect definition of functional modules.

A physical interaction often indicates a functional relation, we therefore next combine our module definition with Tandem Affinity Purification (TAP) data, which is the base of our two high-throughput derived module datasets [14],[15]. We use the mean Purification Enrichment (PE) score [15] between component pairs for each module in order to quantify the average propensity of its constituents to (indirectly) interact. We restrict this analysis to complexes, because physical interactions are biologically most relevant in that context and many pathway components (i.e. metabolic enzymes) do not have any interaction partner in our TAP dataset.

Cohesive complexes have a higher mean PE score than flexibly evolving ones, but this observation is biased towards the multitude of complexes that are automatically generated from high throughput interaction data (P = 0.017, table 5a in Text S1). If we look at the curated complex datasets separately, results point in a different direction. Much to our own surprise, cohesive modules from curated datasets tend to have a *lower* mean PE score than flexibly evolving ones from the same dataset (P = 0.001, table 5a in Text S1). Similarly, removal of subunits which are most loosely attached to the rest of the complex, has a small and mixed effect on evolutionary cohesiveness (table 5b in Text S1).

If we remove subunits which most likely interaction partner is not part of the same module, we observe no significant increase in cohesiveness (table 5d in Text S1). A strong interaction of a module component with a protein outside the module apparently does not indicate that this component is not part of the module, or that it has an additional function outside the module. On the contrary: it may be its function within the module to interact with other parts of the system. These results indicate that a probable physical interaction is neither a necessary nor sufficient condition for a functional relation. Even given the fact that the TAP experiments are not exhaustive with respect to different growth conditions and there are probably many more interactions than those we know about, it is clear that functional relations extend physical ones.

### Orthologs and Inparalogs

We have established that reducing the conceptual and technical ambiguity in functional module definition increases the observed evolutionary cohesiveness. Now we test the robustness of our results to the definition of orthologous groups. We run orthoMCL [16] with default parameters on our set of species. Using this orthology as sole data source, the fraction of cohesive modules and the average cohesiveness scores are qualitatively the same as when we use our KOG-based orthology assignments (table 6a in Text S1).

More importantly, we can cross-compare our original KOG-based orthologous groups with the groups defined by the orthoMCL method. If we trust only those orthologous groups with 90% overlap with an orthoMCL group, removing the unreliable orthologous groups results in an increase in cohesiveness, except for the datasets which contain large modules: KEGG and the socio-affinity clusters. The orthologous groups deemed unreliable typically contain more species than the trusted ones (P = 0.0). Discarding unreliable orthologous groups means we remove components which are present in many species, which, within our scoring scheme, has more negative impact on the evolutionary cohesiveness of large modules than of small modules. If we compare submodules to original modules we find no significant increase in cohesiveness, except for the datasets derived from high-throughput experiments (table 6b in Text S1). However, the overall fraction of cohesiveness increases from 27% to 31% (Figure 3 and table 6c in Text S1), an increase which mainly results from removing modules which consist solely of unreliable KOGs. As was the case with module definitions, we observe that a more conservative definition of orthologous groups results in a higher degree of cohesiveness.

Apart from obvious problems with incorrect assignments, which we tried to tackle by cross filtering with orthoMCL, there are more ways in which the use of orthologous groups to infer presence and absence of module components in different genomes distorts the quantification of cohesive evolution. A module which is completely absent in a certain species could have retained a functionally differentiated recent duplicate of one of its components. In the phylogenetic profile this would correspond to a column of all zeros and a one, while the actual module is completely missing. This phenomenon of functional differentiation is more likely to occur as a family has more duplications and we expect that components of cohesive modules are generally assigned to orthologous groups with few inparalogs.

We find that indeed cohesively evolving modules tend to be composed of orthologous groups which contain few inparalogs (P = 5e-07, table 7a in Text S1). We adopt the approach described by Snel et al. [9] and remove the 50% orthologous groups containing most inparalogs from our datasets. The resulting submodules evolve more cohesively than the original modules (P = 0.02, table 7b in Text S1) and the fraction of cohesive modules across all datasets increases from 27% to 33% (table 7c in Text S1). Datasets which comprise mainly of large modules do not show an increase in cohesiveness. We can explain this by the fact that large modules are often distinctively cohesive by virtue of being completely present in a large number of species. Removing presence which is possibly but not necessarily spurious, is therefore not likely to increase the measured evolutionary cohesiveness in large modules.

The paralogy filter strongly suggests that on the level of protein families, functional divergence is likely to be one of the factors influencing evolutionary cohesiveness. However, whether this caused by the fact that sometimes a functionally diverged duplicate is present, while a duplicate which retained the original function is lost, or whether it is the case that large families typically are not part of cohesive modules, remains debatable.

We tested multifunctionality on the level of individual proteins by integration of high throughput and literature derived functional information (Text S2). However, we have not been able to show convincingly that multifunctionality of a protein plays an important role in explaining the observed evolutionary flexibility.

### Cohesively versus Flexibly Evolving Functional Modules and Pathways versus Complexes

Given the fact that some modules evolve cohesively and others do not, one of the questions we want to answer is whether, and if so, in what respects cohesively evolving modules are different from flexibly evolving modules. Cohesively evolving modules tend to have a lower rate of sequence evolution (P = 0.0009, comparing Dn/Ds rates from [17] of cohesively versus flexibly evolving modules), reflecting that they're subject to stronger negative selection pressure. As mentioned above, components of cohesively evolving modules tend to duplicate less often than components of flexibly evolving modules. We compared the average propensity of module components to interact with each other between cohesively and flexibly evolving modules. We found to our own surprise, that for the curated complex datasets, components of cohesively evolving complexes actually were less likely to interact among each other than components of flexibly evolving complexes.

Another interesting question is whether cohesive evolution is more likely to occur in certain biological processes than others. We detect overrepresented Gene Ontology (GO) categories [18] of proteins in cohesive modules with respect to all proteins in functional modules using the BiNGO plugin in Cytoscape [19]. (figure 8 in Text S1 and Tables S1, S2, S3). Proteins which are part of cohesively evolving modules are involved in core processes: amino acid metabolism, protein ribosome biogenesis, electron transport and generation of precursor metabolites and energy.

It may be the case that modules engaged in these essential processes are not particularly cohesively evolving, but just very conserved. A comparison of the number of species assigned to KOGs containing cohesively evolving module components assigned to these overrepresented GO categories, to a background of all KOGs shows that indeed proteins involved in translation, cytoplasm organization and biogenesis, ribosome biogenesis and assembly are more conserved than the background. In contrast, proteins involved in the other overrepresented core processes such as, for example, amino acid metabolism, are less conserved compared to the background of all module components (Table S1). This shows that there are in fact modules which do not evolve cohesively only because all components are essential (and therefore conserved). These modules are mainly involved in core metabolic processes.

The overrepresentation of metabolic GO categories among cohesively evolving modules corresponds to a striking difference in cohesiveness observed between datasets containing complexes, and pathway datasets (Table 2). Biochemical pathways evolve more cohesively than complexes (P = 0.00012 comparing pathways with curated complexes, P<1e-100 comparing pathways to all complexes). In fact, whether a module is a pathway or a complex, is a good predictor for cohesive evolution (figure 3 and table 3a in Text S1). The difference between pathways and complexes is more significant among small modules, which distinguish themselves from the random background by being completely absent in multiple species (table 3b in Text S1).

## Discussion

The present study is the first large scale investigation of cohesive evolution of functional modules in eukaryotes. We show similar evolutionary behavior of functional modules in eukaryotes to what is previously observed for prokaryotes: most modules evolve flexibly [9]–[11] and curated modules evolve more cohesively than modules derived from high throughput interaction data [9]. As eukaryotes do not contain operons that facilitate the simultaneous loss of module components, all cohesive evolution that we observe is the result of nongenomic properties of the functional module. Hence the system level properties of functional modules are important in the cohesive loss of subunits. Nonetheless a substantial level of flexibility seems resistant to conceptual and technical filtering.

We attempt to estimate the relative importance of mistakes in the definition of functional modules and the use of orthologous groups to determine presence and absence of module components in our set of genomes. We increase reliability, both of our set of functional modules as well as our set of orthologous groups and find that cohesiveness is increased with approximately 30%. Removing orthologous groups which are likely to have functionally differentiated also increases the fraction of cohesive modules with ∼30%.

Ideally, we want to overlay all those filters on top of each other, but if we do, we remove so many modules and module components that we are left with less than 13% of our original number of modules and the modules which remain are typically very small (2 or 3 components). Even after application of all these filters we still observe that most functional modules do not evolve more cohesively than random (46% of modules have a cohesiveness score>0.99). (Figure 3).

Naturally, our approach has some limitations in capturing and classifying the diversity in possible evolutionary scenario's illustrated by manually curated examples [2]–[8], (Text S2). The assignment of proteins to orthologous groups is neither exhaustive nor completely correct and not all of the mistakes can be filtered out. Moreover, there are many other ways in which proteins co-evolve (similar rate [20], compensatory mutations [21], coduplication [22]) and our cohesiveness score is restricted to cooccurrence only. These limitations, inherent in a large scale analysis, also apply to the use of phylogenetic profiles to determine functional relations between pairs of proteins. Recent evaluations of phylogenetic profiling methods show that reliable results are obtained at a cost of very low sensitivity, especially in eukaryotes [23]. The evaluated methods are more advanced than simply counting co-presence and co-absence. Nevertheless either many functionally related pairs are not detected or many unrelated pairs are being classified as coevolving. Strikingly, the related pairs which can be reliably retrieved belong to functional classes representing those cellular processes, which are fundamental for any cell in any kingdom of life, which corresponds to what we have observed in this study.

Manual reconstructions of the evolution of functional modules, complex purification with missing subunits across different species [24], as well as previous large scale investigation of evolutionary cohesiveness of functional modules and the evaluations of phylogenetic profiling methods all point into the same direction. Therefore, even though the exact degree of coevolution is probably underestimated, we conclude that functionally related proteins do not necessarily coevolve, and functional modules do not need to behave as evolutionary modules.

## Methods

### Module Datasets

We obtained the SGD pathway dataset from the *Saccheromyces* Genome Database (ftp://genome-ftp.stanford.edu/pub/yeast/data_download/literature_curation/biochemical_pathways.tab), the KEGG pathway datasets from the KEGG website (ftp://ftp.genome.jp/pub/kegg/pathway/organisms/sce/sce_gene_map.tab and ftp://ftp.genome.jp/pub/kegg/pathway/map_title.tab), the socio-affinity clusters were provided in the Supplementary Information of the publication [14] and the Purification Enrichment clusters were obtained from personal communication. The MIPS dataset was downloaded from ftp://ftpmips.gsf.de/yeast/catalogues/complexcat [25] and the Aloy dataset from http://www.russell.embl.de/complexes/ [26].

We deleted per dataset the modules of which a submodule was also present in that dataset. We deleted from the pathway datasets those modules which were complexes rather than pathways (SGD pathways: pyruvate dehydrogenase, KEGG pathways: Ribosome, Proteasome, DNA polymerase, RNA polymerase). Modules from which components are assigned to one orthologous group, as well as modules which consist of only one protein or for which we could only map one protein to a systematic ORF name were excluded. Mapping to systematic ORF names was done via the gene registry file from SGD (ftp://genome-ftp.stanford.edu/pub/yeast/gene_registry/registry.genenames.tab).

### Orthologous Groups

Due to dynamics of protein evolution such as protein fusion, protein fission and domain acquisition and loss, defining orthologous groups is a nontrivial task. Therefore we choose a set of well-established, manually curated orthologous groups from the KOG database [13] as our starting point. A set of 34 eukaryotic species (Figure 1), including metazoa, amoebazoa, alveolates, excavata and plantae, is selected based on completeness and quality of annotation of their genomes, yielding a total of 368358 proteins. We perform all against all Smith Watermann with Paralign [27] on the protein sequences from the selected species and ran Inparanoid [28] with default parameters (except that we used a threshold on the score rather than on the E-value) on this data for each pair of species. Proteins within one Inparanoid cluster which are from different species are connected with an edge, resulting in a graph connecting 237538 proteins. First, we assign 162250 proteins to pre-existing KOGs from the KOG database [13] with a KOGnitor script. Subsequently, each unassigned protein connected to at least two proteins which are assigned to the same KOG and have an edge between them is assigned to that KOG. This leaves us with 206108 unassigned proteins. In our large graph we identify triangles (trios of interconnected proteins), if a triangle has two components assigned to the same orthologous group, we assign the third component to that group as well. In this way, another 14555 proteins were added to pre-existing KOGs. The remainder of triangles we clustered into 5704 novel orthologous groups using CFinder [29], a program which implements the clique percolation method to detect clusters of fully connected subgraphs of different sizes (in this case size three). We have assigned 214342 out of 368358 proteins to a total of 10548 orthologous groups, more than half of which is novel.

We defined an alternative for our orthologous groups by running the orthoMCL program [16] with default parameters on our set of genomes. We assign a total of 275953 proteins to 40239 orthologous groups.

### Module Definition Filters

For our cross-comparison filter we check for each dataset, for each module, whether there is (complete or partial) overlap with another module in another dataset. If a module is not completely confirmed, we remove unconfirmed subunits such that we keep the largest overlap we have encountered in other datasets.

In order to filter the functional modules with high throughput interaction data, we use the Purification Enrichment (PE) score from [15]. This score integrates data from two large scale interaction (TAP/MS) studies [14],[30]. Both presence and absence of associations are taken into account to derive a measure denoting the likelihood two proteins directly or indirectly interact (see [15] for further detail). We downloaded these PE scores from http://interactome-cmp.ucsf.edu/ on February 23, 2008. For our PE score filter, we only consider modules which components have at least one interaction with another protein (within the module or outside) with a confidence score higher than 0.2 [15]. We first remove all components with a zero PE score with all other module components and cluster the remaining components with single linkage with −1*PE as distance. We obtain two clusters and remove the smallest cluster.

## Supporting Information

Table S1 Overrepresented Gene Ontology Biological Process categories
(0.04 MB XLS)

Table S2 Overrepresented Gene Ontology Molecular Function categories
(0.01 MB XLS)

Table S3 Overrepresented Gene Ontology Cellular Component categories
(0.02 MB XLS)

Text S1 Detailed description of results of different filters applied to functional modules
(1.22 MB PDF)

Text S2 Detailed examples of 5 different complexes and pathways from curated datasets, illustrating different cohesiveness scores and module filters.
(0.20 MB PDF)
