# Supplementary material for: Cohesive versus Flexible Evolution of Functional Modules in Eukaryotes
Source: PLoS Comput Biol. 2009 Jan 30;5(1):e1000276. doi: 10.1371/journal.pcbi.1000276 (PMC2615111; doi:10.1371/journal.pcbi.1000276)
Supplement: Text S1 — Detailed description of results of different filters applied to functional modules (1.22 MB PDF) [file pcbi.1000276.s004.pdf]

Supplementary Material

'Cohesive versus flexible evolution of functional modules in Eukaryotes'

Like Fokkens and Berend Snel

TABLE OF CONTENTS

1: Comparison of average cohesiveness score per dataset for different scoring schemes 3
2: Cohesiveness scores and number of module components 4
3: Pathways compared to complexes 5
4: Cross-comparison with other module datasets 7
5: Cross-validation with TAP data 10
6: Trusted KOGs: comparison with orthoMCL 13
7: Inparalogs 15
8: Overrepresented Gene Ontology Categories 17
References 19

S3 (Overrepresented Gene Ontology Biological Process categories), S4 (Overrepresented Gene Ontology Molecular Function categories) and S5 (Overrepresented Gene Ontology Cellular Component categories) are available in separate excel files.

Supplementary Examples E1 through E5 are available in a separate .pdf file.

The fasta files containing all protein sequences used in this study, as well as the functional modules, the KOGs and the orthoMCL groups can be downloaded at bioinformatics.bio.uu.nl/like/suppl/

1: Comparison of average cohesiveness score per dataset for different scoring schemes

	Avg Co-occurrence	Avg deviation from modular	Homogeneous columns	Species Absent	Species Present	Species Absent, Species Present
SGD	0.82	0.83	0.81	0.59	0.75	0.95
KEGG	0.87	0.87	0.77	0.34	0.74	0.85
PE	0.6	0.6	0.57	0.39	0.53	0.79
socio-affinity	0.74	0.74	0.69	0.14	0.69	0.78
MIPS	0.67	0.67	0.64	0.37	0.57	0.8
Aloy	0.71	0.71	0.64	0.35	0.6	0.77
all	0.7	0.7	0.66	0.3	0.63	0.8
all curated	0.76	0.76	0.71	0.4	0.66	0.84

Table 1. Average score for different datasets and different scoring schemes.
Average Co-occurrence: for each pair of module subunits we calculate the fraction of species in which both subunits are either present or absent together. We average over all component pairs to obtain a score per module.

Average deviation from modular: the sum of the deviation of the number of components of the functional module for each genome to the average number of module components per genome. Adopted from Snel et al. (2004)[1].
Homogeneous Columns: the number of species in which a module is either completely present or completely absent. Adopted from Gavin et al. (2006)[2].
Species Absent, Species present: the number of species in which a module is completely absent and the number of species in which the module is completely present. The vector containing those two scores is the raw score which is used throughout the article.

2: Cohesiveness scores and number of module components

	Avg Co-occurrence	Avg deviation from modular	Homogeneous columns	Species Absent	Species Present	Species Absent, Species Present
Spearman r	0.37	0.38	0.26	-0.4	0.27	0.0045

Table 2. Correlation of cohesiveness score with module size (number of components).
The score used in this article is the only one which does not correlate with the number of subunits in a module, because it consists of both the number of species in which a module is completely absent, as well as the number of species in which a module is completely present. All one dimensional scores, except the number of species in which a species is completely absent, correlate positively with size: modules with many subunits tend to evolve more cohesively according to these scores. The same trend is reported by Campillos et al. (2006) [3], who use a two-dimensional score consisting of the number of evolutionary events (gain or loss) and the number of shared events. We use Spearman rank correlation because because both variables are not normally distributed.

3: Pathways compared to complexes

Table 3a Being a pathway or a complex as a predictor for evolutionary cohesiveness.
Table 2 in the main text and Table 1in the Supplementary text suggest that pathways evolve more cohesively than complexes. We tested this using a Mann Whitney Wilcoxon rank sums test, comparing pathways to curated complexes. We find that pathways indeed tend to have a higher cohesiveness score than complexes and that this difference is significant (average score pathways: 0.9, complexes 0.8, P value 0.00012).

If we would use the categories 'pathway' and 'complex' to predict whether a module is cohesively evolving or not we would get contingency tables like this:

pathways vs all complexes

	pathway	complex	total
cohesive	82	267	349
not cohesive	110	826	936
total	192	1093	1285

P value Fisher exact test: 3.07e-07

curated datasets only:

	pathway	complex	total
cohesive	82	83	165
not cohesive	110	172	282
total	192	255	447

P value Fisher exact test: 0.018

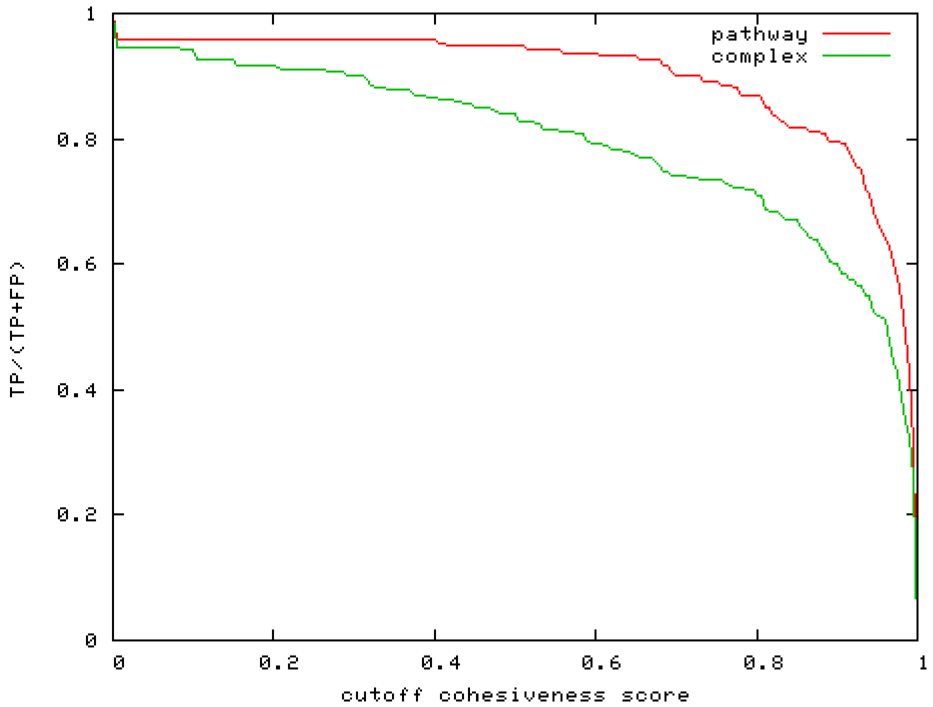

Figure 3 Precision or Positive Predictive Value of pathway (red) and complex (green) categories.
This figure shows that regardless of the specific cohesiveness score cutoff used to classify modules as cohesive or incohesive, the proportion of pathways which is cohesively evolving, is higher than of complexes.

BIN	P value	Number of pathways in bin	Number of complexes in bin
2 – 3	0.005	57	117
4 – 6	0.03	50	60
7 – 10	0.09	33	36
11 – 15	0.07	21	23
>= 16	0.13	31	19

Table 3b Pathways versus complexes for different module sizes
We compared evolutionary cohesiveness for pathways and complexes for different size bins (extra small (2-3 components), small (4-6 components), medium (7-10 components), large (11-15 components) and extra large (>=16 components)). We used a Mann Whitney Wilcoxon rank sums test to determine whether pathway evolve more cohesively than complexes for each size bin.

4: Cross-comparison with other module datasets

	P value ranksums test	N confirmed	Average size
SGD	**0.007**	56	4.56
KEGG	0.193	3	14.89
MIPS	**0.019**	112	5.91
Aloy	0.114	63	6.95
PE	**0.039**	134	4.37
Socio-affinity	0.140	32	11.15
all (nr)	**8.09E-06**	311 (out of 1285 unique modules)	7.74

Table 4a. Scores of confirmed modules compared to scores of unconfirmed modules.
We perform a Wilcoxon rank sums test to compare the distribution of scores of confirmed modules to unconfirmed modules. P values are shown for a one-tailed test: we test whether confirmed modules have higher scores than unconfirmed modules. Confirmed modules are evolving significantly more cohesively than unconfirmed modules in the SGD, PE and MIPS datasets, which are the datasets containing on average the smallest modules. Only a small fraction of the modules in the KEGG pathways and Socio-affinity clusters have been confirmed by other datasets, which may explain why the difference between confirmed and unconfirmed modules is not significant for these datasets.

	P value Wilcoxon T test	N submodules
SGD	0.487	41
KEGG	**0.045**	64
MIPS	0.489	42
Aloy	0.078	22
PE	0.282	107
Socio-affinity	**0.006**	317
all (nr module-submodule combinations)	**0.0009**	593

Table 4b. Scores of confirmed submodules compared to scores of the original, partially confirmed modules. Subunits which have not been confirmed by other datasets are potentially false additions to a module and removal could increase the evolutionary cohesiveness of the module. We compare the cohesiveness score of each completely confirmed submodules with the score of its original module and find that in general this score improves. (Wilcoxon matched pairs test, one tailed: testing whether submodules score higher than the original modules). This difference is more significant for datasets contain large modules (KEGG, Socio-affinity clusters) as there are more submodules to compare in these datasets (see also table 4c below).

CONFIRMED	Fraction cohesive		Average score		N modules			module size	
	no filter	confirmed	no filter	confirmed	N before filter	N after filter	N sub-modules	Average size	Average size after filter
SGD	0.44	0.53	0.95	0.96	106	91	37	4.56	4.02
KEGG	0.38	0.45	0.85	0.93	92	67	55	14.89	5.22
MIPS	0.33	0.38	0.8	0.85	199	151	42	5.91	5.42
Aloy	0.31	0.34	0.77	0.79	87	85	22	6.95	6.6
PE	0.21	0.26	0.79	0.8	433	241	107	4.37	4.93
Socio-affinity	0.24	0.36	0.78	0.83	461	349	309	11.15	6.22
all	0.27	0.36	0.8	0.84	1285	901	497	8.02	5.71

Table 4c. Fraction of modules which evolves cohesively, average score, average size and number of (sub) modules before and after the cross-comparison filter. This filter has less effect on the curated datasets than on the high-throughput data derived module definitions. The increase in the fraction of cohesive modules is the combined effect of an increase in cohesiveness by removing subunits which do not co-occur with the rest of the module in any other dataset and by removing entire unconfirmed modules. All numbers are based on non-redundant module sets: no set of KOGs occurs more than once, except as a sub- or superset.
The high-throughput datasets improve because of cross-comparison with the curated complex sets. The pathway datasets also show a substantial increase in cohesiveness after the filter. Probably this is because pathways are often defined as a set of reactions starting from or ending with a common substrate. Cross-comparison with other datasets may prune a pathway such that only one path between substrates is left.

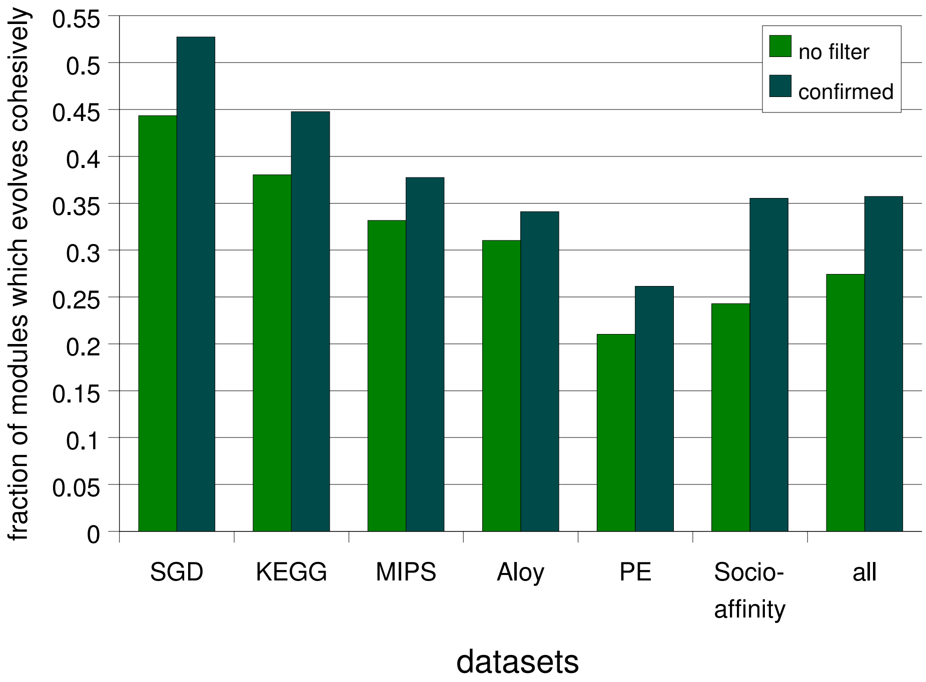

Figure 4. Bar chart of fraction of cohesively evolving modules before and after the cross-comparison filter.

5: Cross-validation with TAP data

Compare average/median/variance PE scores of cohesive modules with average/median/variance PE scores of noncohesive modules

One tailed P value

	P value ranksums		
**datasets**	average PE	median PE	variance PE
PE	**0.016**	**0.009**	0.499
Socio-affinity	**0.021**	0.168	***0.009***
MIPS	***0.026***	***0.037***	0.209
Aloy	*0.085*	*0.205*	0.106
All (nr)	**0.040**	0.097	**0.080**
curated (nr)	***0.009***	***0.032***	0.070
high throughput (nr)	**0.017**	**0.041**	***0.031***

Italics mean/median*:* cohesive lower than noncohesive

Italics variance*:* cohesive higher than noncohesive

Bold: P value<0.05

Table 5a. Average PE score of cohesively evolving modules compared to average PE score of flexibly evolving modules.

We perform a Mann Whitney Wilcoxon rank sums test to test whether subunits of cohesively evolving modules are more likely to interact within the module than subunits of flexibly evolving modules. For the high-throughput datasets, cohesive modules have components which are more likely to interact than those of flexibly evolving modules. For curated datasets however, it is the opposite.

nr: non-redundant: a set of KOGs only occurs once, but may occur as a subset of an other modules set of KOGs.

**datasets**	P value Wilcoxon T test	N submodules
PE	0.173	195
Socio-affinity	**6.25E-005**	235
MIPS	0.203	62
Aloy	0.382	53
All (nr)	**0.029**	546

Table 5b. Scores of submodules compared to scores of the original modules.

Subunits which have no or a very low PE score with other module components are removed (we refer to SE1-SE5 for examples). We compare the cohesiveness score of each submodule with the score of its original module and find that in general this score improves, but, except for the Socio-affinity dataset, this difference is not significant (one-tailed Wilcoxon matched pairs test).

SUB-CLUSTERS PE	Fraction cohesive			Average score			N modules			module size	
	no filter	PE data for all subunits	sub-clusters	no filter	PE data for all subunits	sub-clusters	N with PE data	N after filter	N sub-modules	Average size modules with PE data	Average size after filter
SGD	0.44	0.33	0	0.95	0.82	0.9	6	1	1	3.17	8
KEGG	0.38	0	x	0.85	0.99	x	1	0	0	2	x
MIPS	0.33	0.37	0.32	0.8	0.83	0.86	104	95	59	4.38	3.86
Aloy	0.31	0.29	0.32	0.77	0.77	0.81	76	75	53	6.18	5.45
PE	0.21	0.21	0.22	0.79	0.79	0.81	433	404	195	4.37	3.99
Socio-affinity	0.24	0.24	0.25	0.78	0.78	0.8	322	275	234	8.92	8.9
all	0.27	0.24	0.24	0.8	0.79	0.81	863	771	497	6.28	5.95

Table 5c. Fraction of modules which evolves cohesively, average score, average size and number of (sub) modules (1) before any filter (no filter), (2) for modules for which all subunits have at least one interaction (not necessarily within the module) a PE score with confidence > 0.2 [4] (PE data for all subunits) and (3) after the filter (subclusters). First we remove all components which have a zero PE score with all other module subunits. Subsequently we cluster the module subunits with single linkage clustering, using PE scores as a similarity metric. We obtain two clusters and remove the smallest cluster from the module.

The pathway datasets have very few modules for which all components interact with at least one other protein. Metabolic proteins typically interact via their substrates or not at all. Hence any interactions will be transient at best and are less likely to be picked up by TAP experiments. All numbers are for non redundant sets: no set of KOGs occurs twice in a dataset, but may occur as a subset. The number of submodules reported in table S5b may exceed the number reported here because in table 5b we consider unique original module – submodule combinations. Two modules, when pruned, can yield the same submodule, which would be counted twice in table S4b and only once in this table.

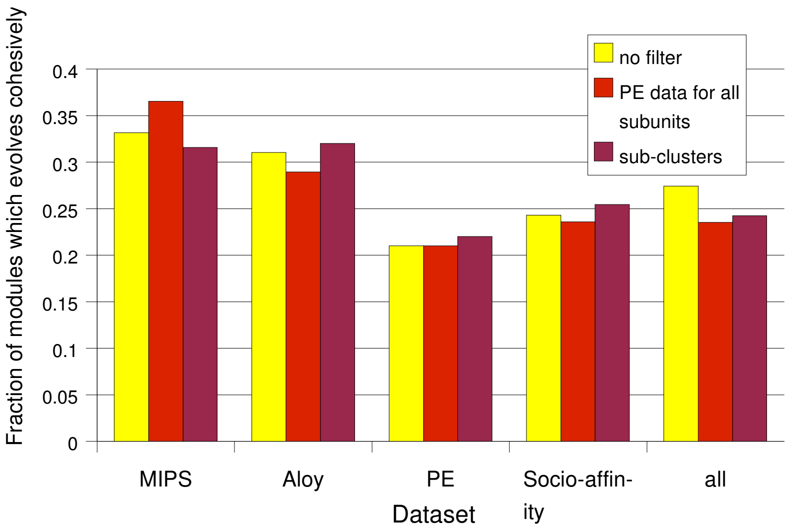

Figure 5. Bar chart of fraction of cohesively evolving modules before and after the filter.

If we compare the average/median/variance PE scores within the module, before and after applying the filter, we find that the average and median PE score increase significantly after the filter (P value 0.0003 and 0.003 respectively, P value from one tailed Wilcoxon rank sums test) and the variance decreased, but not significantly (P value 0.068). Cohesiveness in terms of interaction is significantly increased after this filter, however, the evolutionary cohesiveness is not.

	Fraction cohesive			Average score			N modules			module size	
	no filter	PE data for all subunits	highest PE inside module	no filter	PE data for all subunits	highest PE inside module	N with PE data	N after filter	N sub-modules	Average size modules with PE data	Average size after filter
SGD	0.44	0.33	0	0.95	0.82	0.91	6	1	1	3.17	7
KEGG	0.38	0	x	0.85	0.99	x	1	0	0	2	x
MIPS	0.33	0.37	0.35	0.8	0.83	0.82	104	95	27	4.38	4.46
Aloy	0.31	0.29	0.29	0.77	0.77	0.78	76	75	23	6.18	5.71
PE	0.21	0.21	0.23	0.79	0.79	0.8	433	404	72	4.37	4.44
Socio-affinity	0.24	0.24	0.18	0.78	0.78	0.79	322	275	170	8.92	6.37
all	0.27	0.24	0.22	0.8	0.79	0.79	863	771	284	6.28	5.29

Table 5d. Filter out those subunits who are more likely to interact outside the module than with an other component within the module.

The filter removes all subunits which have a higher PE score with a protein which is not a part of the module than with any other module subunit. This table lists the fraction of modules which evolves cohesively, the average score, the average size and number of (sub) modules (1) before any filter (no filter), (2) for modules for which all subunits have a PE score with confidence > 0.2 (not necessarily with an other module subunit) (PE data for all subunits) and (3) after the filter (highest PE inside module). All numbers are for non redundant sets: no set of KOGs occurs twice in a dataset, but may occur as a subset. Despite modules being pruned, the average size for modules in a dataset can increase, because some modules completely disappear because all their subunits interact more strongly outside the module than with their fellow subunits.

6: Trusted KOGs: comparison with orthoMCL

	Fraction cohesive		Average score	
DATASET	KOG	orthoMCL	KOG	orthoMCL
SGD	0.44	0.41	0.95	0.9
KEGG	0.38	0.33	0.85	0.72
MIPS	0.33	0.36	0.8	0.82
Aloy	0.31	0.36	0.77	0.83
PE	0.21	0.19	0.79	0.81
Socio-affinity	0.24	0.18	0.78	0.75
all	0.27	0.24	0.8	0.79
all curated	0.37	0.37	0.84	0.82

Table 6a. Fraction of modules which evolve cohesively and average score for modules composed of orthologous groups based on KOG and modules composed of orthologous groups obtained by running orthoMCL [5]. Datasets containing large modules (KEGG and Socio-affinity) score a bit lower when subunits are assigned to orthoMCL orthologous groups than when subunits are assigned to KOG groups. The average module size per datasets remains qualitatively the same. Large modules evolve more cohesively than the random background because the module is present entirely in many species. Apparently, the random background of orthoMCL groups contains more groups which are conserved in many species.

	P value Wilcoxon T test	N sub-modules
SGD pathways	***0.04***	47
KEGG	0.08	67
PE clusters	**0.002**	127
Socio-affinity clusters	**0.004**	292
M.I.P.S.	0.06	87
Aloy et al.	0.051	49
all (non redundant)	0.08	136

Table 6b. Scores of submodules without any unreliable orthologous groups compared to scores of the original modules. Scores in **bold**: P value < 0.05, scores in *italics*: scores submodules lower than the scores of the original module. The number of submodules in this table may deviate from the number of submodules mentioned in table S6c. In this table we base our analysis on all unique combinations of original – and submodules. Two modules, when pruned, can yield the same submodule, which would be counted twice in this table and only once in table 6c.

TRUSTED KOGs	Fraction cohesive		Average score		N modules			module size	
	no filter	Trusted KOGs	no filter	Trusted KOGs	N before filter	N after filter	N sub-modules	Average size	Average size after filter
SGD	0.44	0.48	0.95	0.94	106	52	44	4.56	3.13
KEGG	0.38	0.25	0.85	0.85	92	67	67	14.89	8.45
MIPS	0.33	0.44	0.8	0.87	199	109	84	5.91	5.01
Aloy	0.31	0.45	0.77	0.87	87	64	49	6.95	5.31
PE	0.21	0.32	0.79	0.85	433	194	127	4.37	3.98
Socio-affinity	0.24	0.21	0.78	0.76	461	321	291	11.15	6.64
all	0.27	0.3	0.8	0.82	1378	760	610	8.02	5.73

Table 6c. Fraction of modules which evolves cohesively, average score, average size and number of (sub) modules before and after the filter, for which we remove all KOGs which do not have an overlap>90% with any orthoMCL group. This filter makes no distinction between ill defined orthologous groups because of flaws in the algorithm and ill defined groups because the protein family is difficult to characterize (e.g. fast evolving or containing promiscuous domains). Although 'easy' families are unlikely to expose flaws in the procedure of defining orthologs.

All numbers are for non redundant sets: no set of KOGs occurs twice in a dataset, but may occur as a subset.

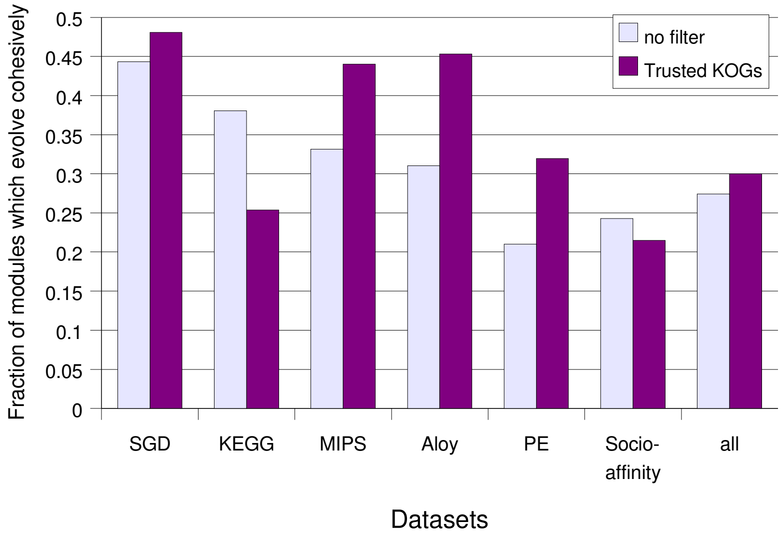

Figure 6. Bar chart of fraction of cohesively evolving modules before and after the filter.

7: Inparalogs

One tailed test	P value ranksums	
**datasets**	N inparalogs	fraction inparalogs
SGD	0.33	0.36
KEGG	**0.02**	**0.04**
MIPS	**0.01**	0.06
Aloy	0.49	0.29
PE	**4.10E-05**	**0.001**
Socio-affinity	**0.004**	0.28
all	**1.50E-06**	**0.004**

Table 7a. Average number resp. fraction of inparalogs of cohesively evolving modules compared to average number resp. fraction of inparalogs of flexibly evolving modules. Fraction of inparalogs: number of inparalogs / number of members of the orthologous group. We perform a Wilcoxon rank sums test to compare the amount of duplications in families constituting cohesive modules with families constituting flexible modules. P values are shown for a one-tailed test: we test whether cohesive modules are comprised of KOGs with fewer inparalogs. To see whether this results depends strongly on the exact measure chosen we also test the difference in *fraction* of inparalogs, which indeed yields less significant results, suggesting that part of the effect of removing KOGs with many inparalogs is due to the fact that these KOGs are conserved in many species.

**datasets**	P value Wilcoxon T test
SGD	0.45
KEGG	0.18
MIPS	**0.0001**
Aloy	0.14
PE	**0.001**
Socio-affinity	0.27
all	**0.02**

Table 7b. Scores of submodules of which KOGs with many inparalogs were removed, compared to scores of the original modules. In the pathway datasets the difference is not significant. In the complex datasets the difference is only significant for those datasets containing smaller modules on average (which are more likely to improve after removing a row containing possible false positives). Scores in **bold**: P value < 0.05.

INPARALOGS	Fraction cohesive		Average score		N			module size	
	no filter	KOGs with many inparalogs removed	no filter	KOGs with many inparalogs removed	N before filter	N after filter	N sub-modules	Average size	Average size after filter
SGD	0.44	0.54	0.95	0.96	106	37	33	4.56	3.11
KEGG	0.38	0.37	0.85	0.83	92	54	53	14.89	9.54
MIPS	0.33	0.5	0.8	0.88	199	110	68	5.91	5.23
Aloy	0.31	0.39	0.77	0.87	87	62	41	6.95	5.74
PE	0.21	0.33	0.79	0.85	433	191	106	4.37	4.21
Socio-affinity	0.24	0.21	0.78	0.78	461	286	256	11.15	6.37
all	0.27	0.32	0.8	0.83	1378	687	518	8.02	5.8

Table 7c. Fraction of modules which evolves cohesively, average score, average size and number of (sub) modules before and after the filter. For this filter we remove the top 50% containing most inparalogs of all KOGs constituting a functional module, boiling down to removing all KOGs with more than 7 inparalogs. Although the improvement of submodules over the original modules was not significant in the pathway datasets (table 7b), the SGD pathway dataset contains a larger fraction of cohesive modules than before the filter. However, this increase comes at a cost: more than 2 third of the modules is removed completely.
All numbers are for non redundant sets: no set of KOGs occurs twice in a dataset, but may occur as a subset.

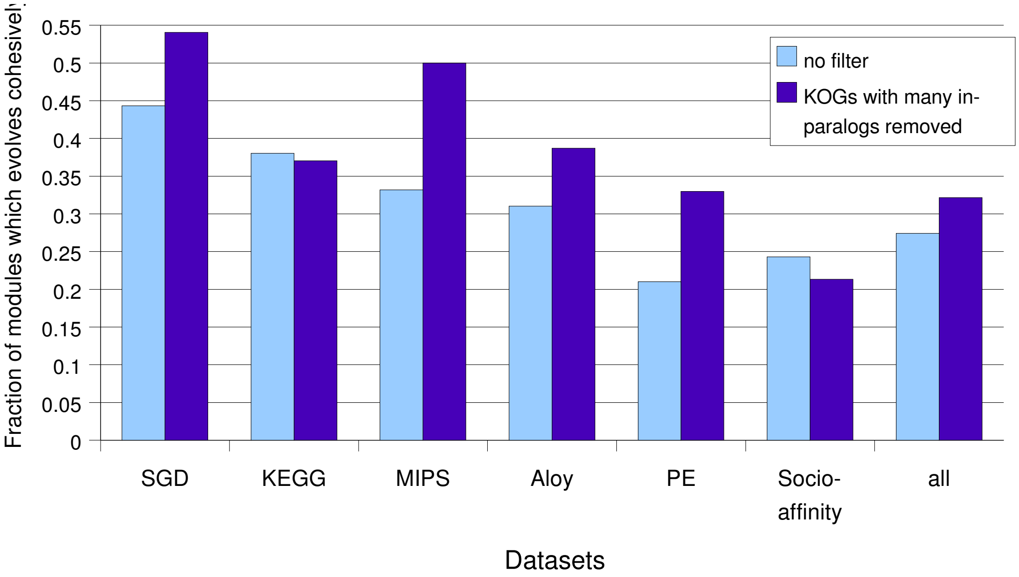

Figure 7. Bar chart of fraction of cohesively evolving modules before and after the filter.

8: Overrepresented Gene Ontology categories

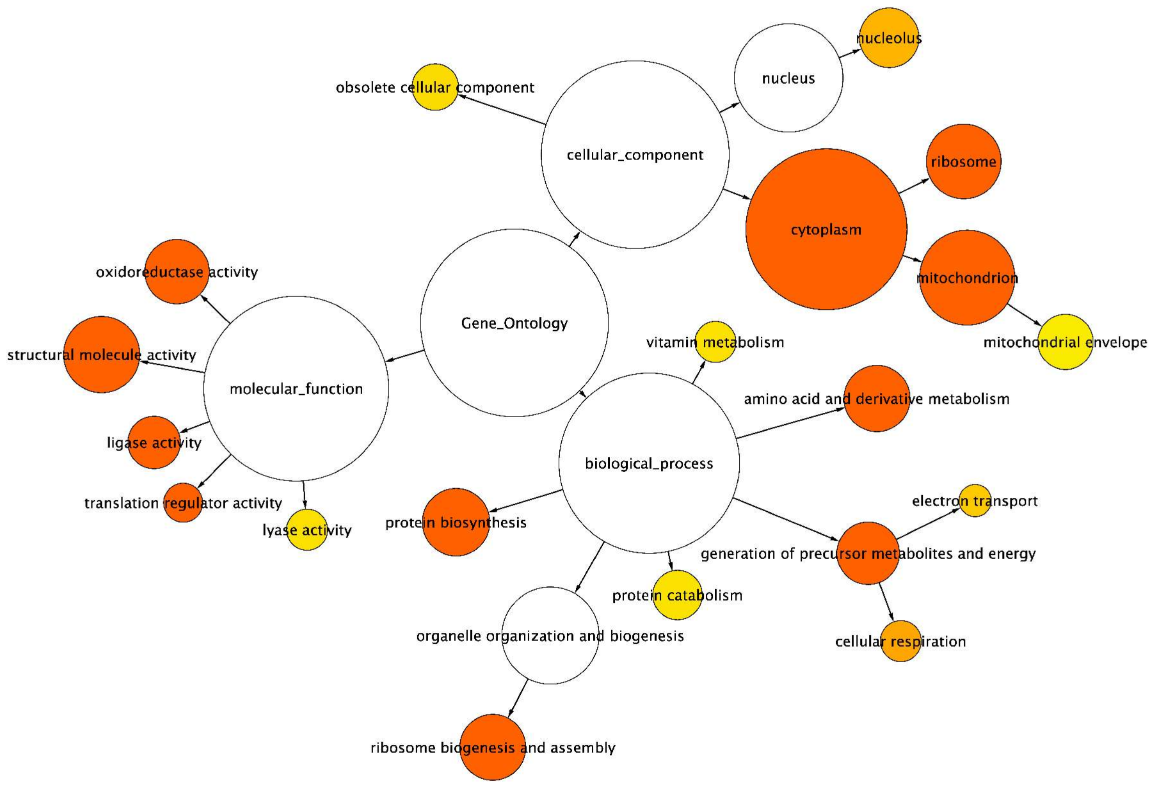

Figure 8 Overrepresentation Gene Ontology categories
This plot is generated with the BiNGO plugin in Cytoscape. It represents the overrepresented GO Slim Yeast categories of proteins constituting cohesively evolving modules with respect to proteins from flexibly evolving modules. The color of the nodes represents the P value (corrected with Benjamini Hochberg correction) of the hypergeometric test ranging from <0.01 (yellow) to < 1E-07 (dark orange).

References

1 Snel B., Huynen M.A. (2004). Quantifying modularity in the evolution of biomolecular systems. Genome Res. 14: 391-397.

2 Gavin AC., Aloy P., Grandi P., Krause R., Boesche M. et al. (2006). Proteome survey reveals modularity of the yeast cell machinery. Nature 440: 631-636.

3 Campillos M., von Mering C., Jensen L.J., Bork P. (2006). Identification and analysis of evolutionarily cohesive functional modules in protein networks. Genome Res. 16: 374-382.

4 Collins S., Kemmeren P., Zhao X-C., Greenblatt J., Spencer F., et al. (2007). Toward a comprehensive atlas of the physical interactome of Saccharomyces cerevisiae. Mol. Cell. Proteomics 6: 439-450

5 Li L., Stoeckert C.J. Jr., Roos D.S. (2003). OrthoMCL: identification of ortholog groups for eukaryotic genomes. Genome Res. 13: 2178-2189.
