# Supplementary material for: Cohesive versus Flexible Evolution of Functional Modules in Eukaryotes
Source: PLoS Comput Biol. 2009 Jan 30;5(1):e1000276. doi: 10.1371/journal.pcbi.1000276 (PMC2615111; doi:10.1371/journal.pcbi.1000276)
Supplement: Text S2 — Detailed examples of 5 different complexes and pathways from curated datasets, illustrating different cohesiveness scores and module filters. (0.20 MB PDF) [file pcbi.1000276.s005.pdf]

Supplementary Examples

'Cohesive versus flexible evolution of functional modules in Eukaryotes'

Like Fokkens and Berend Snel

TABLE OF CONTENTS

E1: A flexibly evolving complex: Nup84 complex (Aloy) 3
E2: A flexibly evolving complex: NPS1 complex (MIPS) 6
E3: A cohesively evolving complex: TFIIH complex (MIPS) 9
E4: A cohesively evolving pathway: Valine biosynthesis (SGD) 12
E5: A flexibly evolving pathway: Valine degradation (SGD) 14
References 16

Supplementary Material S1-S6 is available in a separate .pdf file.

S7 (Overrepresented Gene Ontology Biological Process categories), S8 (Overrepresented Gene Ontology Molecular Function categories) and S9 (Overrepresented Gene Ontology Cellular Component categories) are available in separate excel files.

Fasta files with protein sequences, tab files of KOGs, orthoMCL groups and the functional modules which are used in this study are available for download at bioinformatics.bio.uu.nl/like/suppl/

E1: A flexibly evolving complex: Nup84 complex (Aloy)

Description:
The Nup84 complex, defined in the Aloy dataset, is a subcomplex of the Nuclear Pore Complex (NPC) and consists of 5 nucleoporins and a COPII coat complex subunit. It is involved in the nuclear export of mRNA and plays an important role in the biogenesis of the Nuclear Pore Complex.

Subunits:

ORF	gene name	annotation	KOG
YGL092W	NUP145	Essential nucleoporin, catalyzes its own cleavage in vivo to generate a C-terminal fragment that assembles into the Nup84p subcomplex of the nuclear pore complex, and an N-terminal fragment of unknown function that is homologous to Nup100p	KOG0845
YLR208W	SEC13	Component of both the Nup84 nuclear pore sub-complex and of the COPII complex (Sar1p, Sec13p, Sec16p, Sec23p, Sec24p, Sec31p, Sfb2p, and Sfb3p) which is important for the formation of ER to Golgi transport vesicles	KOG1332
YDL116W	NUP84	Subunit of the nuclear pore complex (NPC), forms a subcomplex with Nup85p, Nup120p, Nup145p-C, Sec13p, and Seh1p that plays a role in nuclear mRNA export and NPC biogenesis	KOG1964
YGL100W	SEH1	Nuclear pore protein that is part of the evolutionarily conserved Nup84p complex (Nup84p, Nup85p, Nup120p, Nup145p, and Seh1p); homologous to Sec13p	KOG2445
YJR042W	NUP85	Subunit of the Nup84p subcomplex of the nuclear pore complex (NPC), required for assembly of the subcomplex and also for formation of the nucleocytoplasmic Gsp1p concentration gradient that plays a role in nuclear trafficking	KOG2271
YKL057C	NUP120	Subunit of the Nup84p subcomplex of the nuclear pore complex (NPC), required for even distribution of NPCs around the nuclear envelope, involved in establishment of a normal nucleocytoplasmic concentration gradient of the GTPase Gsp1p	KOG8539

(annotation from the *Saccheromyces* Genome Database)

Profile:

KOG	*G. lamblia*	*L. major*	*P. falciparum*	*T. parvum*	*C. parvum*	*O. tauri*	*C. merolae*	*A. thaliana*	*O. sativa*	*E. cuniculi*	*R. oryzae*	*C. neoformans*	*U. maydis*	*S. pombe*	*Y. lipolytica*	*C. guillermondii*
KOG0845	1	4	1	2	3	2	1	5	20	3	3	2	2	4	4	3
KOG1332		2	1		1	1	1	2	3	1	1	1	1	1	1	1
KOG1964						1		1	1			1	1	1	1	1
KOG2445								1	1		1	1		1	1	1
KOG2271								1	1			1	1	1	1	1
KOG8539																

KOG	*D. hansenii*	*C. glabrata*	*S. cerevisiae*	*K.lactis*	*E. gossypii*	*F. graminearum*	*N. crassa*	*S. sclerotiorum*	*C. immitis*	*A. nidulans*	*A. terreus*	*A. fumigatus*	*C. elegans*	*D. melanogaster*	*H. sapiens*	*M. musculus*	*D. discoidum*	*E. histolytica*
KOG0845	6	7	11	8	3	3	2	3	4	3	3	2	5	3	3	2	1	1
KOG1332	1	2	1	1	1	1	1	1	1	1	1	1	1	1	1	1	1	1
KOG1964	1	1	1	1	1	1	1	1	1	1	1	1	1	1	1	1	1	
KOG2445	1	1	1	1	1	1	1	1	1	1	1	1	1	1	1	1	1	
KOG2271	1	1	1	1	1								1	1	1	1	1	
KOG8539	1	1	1	1	1													

Cohesiveness Scores:

Scores	raw	compared to background
Avg Co-occurrence	0.62	0.37
Avg Deviation From Modular	0.23	0.41
Homogeneous Columns	5	0.4
Species Absent	0	0
Species Present	5	0.48
Species Present, Species Absent	(5, 0)	0.48

Cohesiveness scores for different measures. Species Present, Species Absent is the score used throughout the article. Raw scores are compared to 100000 random modules of the same size, cohesiveness scores for each raw scores are obtained by counting the number of random modules with a better raw score (or in case of a multidimensional method: better raw scores) and dividing that number by 100000.

Filters:

Cross-comparison of module datasets

This complex occurs in the MIPS dataset as 'Nup84 complex' and in the two high-throughput datasets. The PE clusters dataset contains a module with these components plus two additional subunits: MTR2 and MEX67 which together form a RNA binding mRNA export complex. These two proteins are known to bind the assembled Nup84 complex (Yao et al. 2008). This larger complex has the same raw score and a higher cohesiveness score: 0.59.

The socio-affinity clusters dataset contains a 'Nup84 sub-complex' which consists of all components of the Aloy and MIPS Nup84 complex and has 9 additional subunits: two 'modules' associated with ER to Golgi transport and COPII vesicles (a module consisting of SEC16, SEC23 and SFB2 and a module consisting of SEC24 and GRH1), SEC31, ERO1: a thiol oxidase required for oxidative protein folding in the endoplasmic reticulum, STT3: a subunit of the oligosaccharyltransferase complex of the ER lumen, which catalyzes asparagine-linked glycosylation of newly synthesized proteins and CYS4: a cystathionine beta-synthase, which catalyzes the synthesis of cystathionine from serine and homocysteine. The 'core' complex of this socio-affinity cluster is exactly the same as the Aloy and MIPS Nup84 complex. (For a more detailed explanation of Complexes, Cores and Modules in the socio-affinity dataset, we refer to Gavin et al. 2006). This socio-affinity cluster has a raw score (5,0) like the Aloy Nup84 complex, but a higher cohesiveness score (0.85) due to its large size.

Filtering with Purification Enrichment scores:

	NUP145	SEC13	NUP84	SEH1	NUP85	NUP120
NUP145		6.5	9.8	7.1	6.8	9.4
SEC13	6.5		5.8	6.7	5.0	4.6
NUP84	9.8	5.8		6.4	6.8	9.4
SEH1	7.1	6.7	6.4		4.3	6.7
NUP85	6.8	5.0	6.8	4.3		4.9
NUP120	9.4	4.6	9.4	6.7	4.9	

We first remove all components with a zero PE score with all other components and cluster the components with single linkage with -1* PE score as distance, we obtain two clusters and remove the smallest cluster. The first step does not apply to any of the module constituents, the second step results in removing SEC13 from the module.

Resulting profile:

KOG	*G. lamblia*	*L. major*	*P. falciparum*	*T. parvum*	*C. parvum*	*O. tauri*	*C. merolae*	*A. thaliana*	*O. sativa*	*E. cuniculi*	*R. oryzae*	*C. neoformans*	*U. maydis*	*S. pombe*	*Y. lipolytica*	*C. guillermondii*	*D. hansenii*	*C. glabrata*	*S. cerevisiae*	*K.lactis*	*E. gossypii*	*F. graminearum*	*N. crassa*	*S. sclerotiorum*	*C. immitis*	*A. nidulans*	*A. terreus*	*A. fumigatus*	*C. elegans*	*D. melanogaster*	*H. sapiens*	*M. musculus*	*D. discoidum*	*E. histolytica*
KOG0845	1	4	1	2	3	2	1	5	20	3	3	2	2	4	4	3	6	7	11	8	3	3	2	3	4	3	3	2	5	3	3	2	1	1
KOG1964						1		1	1			1	1	1	1	1	1	1	1	1	1	1	1	1	1	1	1	1	1	1	1	1	1	
KOG2445								1	1		1	1		1	1	1	1	1	1	1	1	1	1	1	1	1	1	1	1	1	1	1	1	
KOG2271								1	1			1	1	1	1	1	1	1	1	1	1								1	1	1	1	1	
KOG8539																	1	1	1	1	1													

The resulting submodule has the same raw score (5,0) and lower cohesiveness score than the original module (0.41) (the distribution of scores of random modules becomes more dispersed as the number of subunits decreases). An other way of filtering with TAP data is to remove those subunits which are more likely to interact with a protein which is not in the module. In this example, SEC13 also fits this criterion: it is most likely to interact with SEC31, which is a component of the COPII coat of secretory pathway vesicles.

Trusted KOGs

We compare our KOG-based orthologous groups with ones obtained with the orthoMCL program and trust only those KOGs who have at least 90% overlap with an orthoMCL orthologous group. This filter removes all orthologous groups except KOG1332 and KOG2445, which results in the following profile:

KOG	*G. lamblia*	*L. major*	*P. falciparum*	*T. parvum*	*C. parvum*	*O. tauri*	*C. merolae*	*A. thaliana*	*O. sativa*	*E. cuniculi*	*R. oryzae*	*C. neoformans*	*U. maydis*	*S. pombe*	*Y. lipolytica*	*C. guillermondii*	*D. hansenii*	*C. glabrata*	*S. cerevisiae*	*K.lactis*	*E. gossypii*	*F. graminearum*	*N. crassa*	*S. sclerotiorum*	*C. immitis*	*A. nidulans*	*A. terreus*	*A. fumigatus*	*C. elegans*	*D. melanogaster*	*H. sapiens*	*M. musculus*	*D. discoidum*	*E. histolytica*
KOG1332		2	1		1	1	1	2	3	1	1	1	1	1	1	1	1	2	1	1	1	1	1	1	1	1	1	1	1	1	1	1	1	1
KOG2445								1	1		1	1		1	1	1	1	1	1	1	1	1	1	1	1	1	1	1	1	1	1	1	1	

This submodule has a raw score (24,2) and an increased cohesiveness score of 0.87.

Inparalogs

We remove orthologous groups which are part of the 50% of orthologous groups with most inparalogs. This filter removes KOG0845 and KOG1332 which leads to a substantial increase in evolutionary cohesiveness: the raw score of the resulting submodule is (5,8) and the cohesiveness score 0.996. Note that in this the filter removes the KOGs to which the two multifunctional proteins (SEC13 and NUP145) in this complex are assigned.

KOG	*G. lamblia*	*L. major*	*P. falciparum*	*T. parvum*	*C. parvum*	*O. tauri*	*C. merolae*	*A. thaliana*	*O. sativa*	*E. cuniculi*	*R. oryzae*	*C. neoformans*	*U. maydis*	*S. pombe*	*Y. lipolytica*	*C. guillermondii*	*D. hansenii*	*C. glabrata*	*S. cerevisiae*	*K.lactis*	*E. gossypii*	*F. graminearum*	*N. crassa*	*S. sclerotiorum*	*C. immitis*	*A. nidulans*	*A. terreus*	*A. fumigatus*	*C. elegans*	*D. melanogaster*	*H. sapiens*	*M. musculus*	*D. discoidum*	*E. histolytica*
KOG1964						1		1	1			1	1	1	1	1	1	1	1	1	1	1	1	1	1	1	1	1	1	1	1	1	1	
KOG2445								1	1		1	1		1	1	1	1	1	1	1	1	1	1	1	1	1	1	1	1	1	1	1	1	
KOG2271								1	1			1	1	1	1	1	1	1	1	1	1								1	1	1	1	1	
KOG8539																	1	1	1	1	1													

E2: A flexibly evolving complex: NPS1 complex (MIPS)

Description:

The NSP1 complex, defined in the MIPS dataset is a subcomplex of the Nuclear Pore Complex (NPC), a large complex which spans the nuclear envelope and enables bidirectional nucleocytoplasmic transport. It is composed of four subunits, of which the NIC96 subunit is embedded in the NPC and is connected to the NUP49-NUP57-NIC96 heterotrimer via its N terminal coiled coil domain (Schrader et al. 2008, Grandi et al. 1995). The translocation through the NPC relies on the interaction of these transport receptors with the FG repeats which protrude from the NUP49-NUP57-NIC96 heterotrimer into the central pore channel. There are multiple copies of this subcomplex residing in the NPC.

Subunits:

ORF	gene name	annotation	KOG
YFR002W	NIC96	Component of the nuclear pore complex, required for nuclear pore formation; forms a subcomplex with Nsp1p, Nup57p, and Nup49p	KOG2168
YJL041W	NSP1	Essential component of the nuclear pore complex, which mediates nuclear import and export	KOG4719<br>KOG2196
YGL172W	NUP49	Subunit of the Nsp1p-Nup57p-Nup49p-Nic96p subcomplex of the nuclear pore complex (NPC), required for nuclear export of ribosomes	KOG0845
YGR119C	NUP57	Nucleoporin, essential subunit of the nuclear pore complex (NPC), functions as the organizing center of an NPC subcomplex containing Nsp1p, Nup49p, Nup57p, and Nic96p	KOG3091

(annotation from the *Saccheromyces* Genome Database)

Profile:

KOG	*G. lamblia*	*L. major*	*P. falciparum*	*T. parvum*	*C. parvum*	*O. tauri*	*C. merolae*	*A. thaliana*	*O. sativa*	*E. cuniculi*	*R. oryzae*	*C. neoformans*	*U. maydis*	*S. pombe*	*Y. lipolytica*	*C. guillermondii*	*D. hansenii*
KOG2168							1	2	1		2	1	1	2	1	1	1
KOG4719	1					1					1	1	1	1	2	1	1
KOG2196	3	1	1	1	1		1	1	1		1	1	1	1	1	1	1
KOG0845	1	4	1	2	3	2	1	5	20	3	3	2	2	4	4	3	6
KOG3091		1						1			2	2	1	1	2	1	1

KOG	*C. glabrata*	*S. cerevisiae*	*K.lactis*	*E. gossypii*	*F. graminearum*	*N. crassa*	*S. sclerotiorum*	*C. immitis*	*A. nidulans*	*A. terreus*	*A. fumigatus*	*C. elegans*	*D. melanogaster*	*H. sapiens*	*M. musculus*	*D. discoidum*	*E. histolytica*
KOG2168	2	1	1	1	1	1	1	1	1	1	1	1	2	1	1	1	
KOG4719	1	3	1	1	1	1	1	1	1				1	5	3		
KOG2196	1	1	6	1	1	1	1	1	1	1	1	1	2	1	3	1	
KOG0845	7	11	8	3	3	2	3	4	3	3	2	5	3	3	2	1	1
KOG3091	1	1	1	1	1	1	1	1	1	1	1	1	1	1	1	1	

Cohesiveness Scores:

Scores	raw	compared to background
Avg Co-occurrence	0.78	0.78
Avg Deviation From Modular	0.14	0.77
Homogeneous Columns	19	0.78
Species Absent	0	0
Species Present	19	0.8
Species Present, Species Absent	(19, 0)	0.797

Cohesiveness scores for different measures. Species Present, Species Absent is the score used throughout the article. Raw scores are compared to 100000 random modules of the same size, cohesiveness scores for each raw scores are obtained by counting the number of random modules with a better raw score (or in case of a multidimensional method: better raw scores) and dividing that number by 100000.

Filters:

Cross-comparison of module datasets

This complex occurs also in the Aloy dataset as 'Nps1 subcomplex of the nuclear pore complex' and passes the cross-comparison filter without losing any subunits.

	NIC96	NSP1	NUP49	NUP57
NIC96		7.3	5.6	4.6
NSP1	7.3		4.6	5.7
NUP49	5.6	4.6		4.8
NUP57	4.6	5.7	4.8	

Filtering with Purification Enrichment scores:

We first remove all components with a zero PE score with all other components and cluster the components with single linkage with -1* PE score as distance, we obtain two clusters and remove the smallest cluster. The first step does not apply to any of the module constituents, the second step results in removing NUP49 from the module.

Resulting profile:

KOG	G. lamblia	L. major	P. falciparum	T. parvum	C. parvum	O. tauri	C. merolae	A. thaliana	O. sativa	E. cuniculi	R. oryzae	C. neoformans	U. maydis	S. pombe	Y. lipolytica	C. guillermondii	D. hansenii	C. glabrata	S. cerevisiae	K.lactis	E. gossypii	F. graminearum	N. crassa	S. sclerotiorum	C. immitis	A. nidulans	A. terreus	A. fumigatus	C. elegans	D. melanogaster	H. sapiens	M. musculus	D. discoidum	E. histolytica
KOG2168							1	2	1		2	1	1	2	1	1	1	2	1	1	1	1	1	1	1	1	1	1	1	2	1	1	1	
KOG4719	1					1					1	1	1	1	2	1	1	1	3	1	1	1	1	1	1	1				1	5	3		
KOG2196	3	1	1	1	1		1	1	1		1	1	1	1	1	1	1	1	1	6	1	1	1	1	1	1	1	1	1	2	1	3	1	
KOG3091		1						1			2	2	1	1	2	1	1	1	1	1	1	1	1	1	1	1	1	1	1	1	1	1	1	

The resulting submodule has a better raw score (19,2) and a higher cohesiveness score than the original module (0.97). An other way of filtering with TAP data is to remove those subunits which are more likely to interact with a protein which is not in the module. This results in removing NSP1, which has the highest PE score (15.1) with NUP82, a nucleoporin and subunit of the Nuclear Pore Complex which forms a subcomplex with NSP1 and NUP159 and is found only on the cytoplasmic periphery of the Nuclear Pore Complex (Ho et al. 2000). The fact that NSP1 is assigned to two orthologous groups (a 'Nuclear pore complex protein' KOG (KOG4719) and a 'Nuclear porin' KOG (KOG2196)) and the fact that it participates in two subcomplexes of the Nuclear Pore Complex, suggest that this is a multifunctional protein.
Removing it from the module results in the following profile:

KOG	G. lamblia	L. major	P. falciparum	T. parvum	C. parvum	O. tauri	C. merolae	A. thaliana	O. sativa	E. cuniculi	R. oryzae	C. neoformans	U. maydis	S. pombe	Y. lipolytica	C. guillermondii	D. hansenii	C. glabrata	S. cerevisiae	K.lactis	E. gossypii	F. graminearum	N. crassa	S. sclerotiorum	C. immitis	A. nidulans	A. terreus	A. fumigatus	C. elegans	D. melanogaster	H. sapiens	M. musculus	D. discoidum	E. histolytica
KOG2168							1	2	1		2	1	1	2	1	1	1	2	1	1	1	1	1	1	1	1	1	1	1	2	1	1	1	
KOG2196	3	1	1	1	1		1	1	1		1	1	1	1	1	1	1	1	1	6	1	1	1	1	1	1	1	1	1	2	1	3	1	
KOG3091		1						1			2	2	1	1	2	1	1	1	1	1	1	1	1	1	1	1	1	1	1	1	1	1	1	

which has a better raw score (23,3) than the original complex but because it has fewer components the cohesiveness score is lower: 0.78 instead of 0.797.

Trusted KOGs

We compare our KOG-based orthologous groups with ones obtained with the orthoMCL program and trust only those KOGs who have at least 90% overlap with an orthoMCL orthologous group. Members of this module are all assigned to untrusted orthologous groups, therefore this filter removes the entire module.

Inparalogs

We remove orthologous groups which are part of the 50% of orthologous groups with most inparalogs. All but one of the orthologous groups associated with this module belong to this top 50% and therefore the filter removes the entire module.

Multifunctional proteins

We have filtered for multifunctional proteins in several ways: one of which was to remove proteins which are also part of an other, non overlapping module in the same module definition. As we have seen in the comparison of PE scores, NSP1 is also part of an other complex containing NUP82 (Grandi et al. 1995). We also filtered for multifunctional proteins with the Gene Ontology. For each module we find overrepresented GO categories with the GO-TermFinder program (http://search.cpan.org/dist/GO-TermFinder/). To each module we assign those categories to which >80% of its subunits are assigned with a corrected P value < 0.001 (hypergeometric test for overrepresentation of GO categories among a group of proteins (subunits of a module)). Then for each subunit, we determine whether it shares the function with the rest of the module, and if so, whether it has an additional function ('parent' or 'child' functional categories are not considered to be different). Those proteins are removed. The fact that we consider only those modules which have the same GO category assigned to 80% of its components, already acts as a powerful filter and the extra benefit from removing multifunctional proteins is very small. In this example, the module has GO categories related to import and export to and from the nucleus associated to it, the proteins NSP1 and NUP49 are also assigned to GO categories related to the import/export/localization of the ribosome. If we remove these two proteins, we get the following profile:

KOG	*G. lamblia*	*L. major*	*P. falciparum*	*T. parvum*	*C. parvum*	*O. tauri*	*C. merolae*	*A. thaliana*	*O. sativa*	*E. cuniculi*	*R. oryzae*	*C. neoformans*	*U. maydis*	*S. pombe*	*Y. lipolytica*	*C. guillermondii*	*D. hansenii*	*C. glabrata*	*S. cerevisiae*	*K.lactis*	*E. gossypii*	*F. graminearum*	*N. crassa*	*S. sclerotiorum*	*C. immitis*	*A. nidulans*	*A. terreus*	*A. fumigatus*	*C. elegans*	*D. melanogaster*	*H. sapiens*	*M. musculus*	*D. discoidum*	*E. histolytica*
KOG2168							1	2	1		2	1	1	2	1	1	1	2	1	1	1	1	1	1	1	1	1	1	1	2	1	1	1	
KOG3091		1						1			2	2	1	1	2	1	1	1	1	1	1	1	1	1	1	1	1	1	1	1	1	1	1	

This profile gets a raw score (14,7) and a cohesiveness score of 0.995, so after removal of multifunctional proteins, this module is evolving cohesively.

E3: A cohesively evolving complex: TFIIH complex (MIPS)

Description:
TFIIH is a transcription factor complex in the MIPS dataset, which, in the presence of ATP, phophorylates RNA polymerase II which allows it to initiate transcription. Phosphorylation is performed by the kinase KIN28.

Subunits:

ORF	gene name	annotation	KOG
YPR056W	TFB4	Component of RNA polymerase transcription initiation TFIIH factor	KOG2487
YLR005W	SSL1	TFIIH subunit (transcription initiation factor), factor B	KOG2807
YDR311W	TFB1	TFIIH subunit (transcription initiation factor), 75 kD	KOG2074
YDL108W	KIN28	Cyclin-dependent ser/thr protein kinase	KOG0659
YDR460W	TFB3	TFIIH subunit (transcription/repair factor)	KOG3800
YPR025C	CCL1	TFIIH subunit (transcription initiation factor), cyclin C component	KOG2496
YER171W	RAD3	DNA helicase/ATPase	KOG1131
YPL122C	TFB2	TFIIH subunit (transcription/repair factor)	KOG3471
YIL143C	SSL2	DNA helicase	KOG1123

(annotation from the *Saccheromyces* Genome Database)

Profile:

KOG	*G. lamblia*	*L. major*	*P. falciparum*	*T. parvum*	*C. parvum*	*O. tauri*	*C. merolae*	*A. thaliana*	*O. sativa*	*E. cuniculi*	*R. oryzae*	*C. neoformans*	*U. maydis*	*S. pombe*	*Y. lipolytica*	*C. guillermondii*	*D. hansenii*
KOG2487			1	1	1	1		1	1	1	1	1	1	1	1	1	1
KOG2807	1	1	1	1	1	1	1	1	1	1	1	1	1	1	1	1	1
KOG2074						1		2	1		1	1	1	1	1	1	1
KOG0659		1	1			1	1	3	1	1	1	1	1	1	1	1	1
KOG3800			1	1	1		1	1		1	1	1	1	1	1	1	1
KOG2496						1	1	1			1	1	1	1	1	1	1
KOG1131	1	1	1	1	1	1	2	1	1	2	1	1	1	1	1	1	1
KOG3471			1	1	1		1	1	1	1	1	1	1	1	1	1	1
KOG1123	1	2	1	1	1	1	1	2	1	1	2	1	1	1	1	1	1

KOG	*C. glabrata*	*S. cerevisiae*	*K.lactis*	*E. gossypii*	*F. graminearum*	*N. crassa*	*S. sclerotiorum*	*C. immitis*	*A. nidulans*	*A. terreus*	*A. fumigatus*	*C. elegans*	*D. melanogaster*	*H. sapiens*	*M. musculus*	*D. discoidum*	*E. histolytica*
KOG2487	1	1	1	1	1	1	1	1	1	1	1	1	1	1	1	1	1
KOG2807	1	1	1	1	1	1	1	1	1	1		1	1	2	1	1	2
KOG2074	1	1	1	1	1	1	1	1	1	1	1	1	1	1	1	1	
KOG0659	1	1	1	1	1	1	1	1	1	1	1	2	2	2	4	1	
KOG3800	1	1	1	1	1	1	1	1	1	1	1	1	1	1	1	1	
KOG2496	1	1	1	1	1	1	1	1	1	1	1	1	1	1	1	10	
KOG1131	1	1	1	1	1	1	1	1	1	1	1	2	1	1	1	1	2
KOG3471	1	1	1	1	1	1	1	1	1	1	1	1	1	1	1	1	2
KOG1123	1	1	1	1	1	1	1	1	1	1	1	1	1	3	1	1	3

Cohesiveness Scores:

Scores	raw	compared to background
Avg Co-occurrence	0.86	0.99
Avg Deviation From Modular	0.09	0.99
Homogeneous Columns	23	0.99
Species Absent	0	0
Species Present	23	0.99
Species Present, Species Absent	(23, 0)	0.99

Cohesiveness scores for different measures. Species Present, Species Absent is the score used throughout the article. Raw scores are compared to 100000 random modules of the same size, cohesiveness scores for each raw scores are obtained by counting the number of random modules with a better raw score (or in case of a multidimensional method: better raw scores) and dividing that number by 100000.

Filters:

Cross-comparison of module datasets

With this filter, we keep the combination of the largest number of components which have been found together in a complex or pathway in one of the other datasets. This module has been completely confirmed by the 'transcription factor TIIFH'-complex in the Aloy dataset, which has two additional components: CDC27 and FPR1.

Additional subunits in 'transcription factor TIIFH'-complex in the Aloy dataset

YBL084C	CDC27	Subunit of the Anaphase-Promoting Complex/Cyclosome (APC/C), a ubiquitin-protein ligase required for degradation of anaphase inhibitors, including mitotic cyclins, during the metaphase/anaphase transition	KOG1126
YNL135C	FPR1	Peptidyl-prolyl cis-trans isomerase (PPIase), binds to the drugs FK506 and rapamycin; also binds to the nonhistone chromatin binding protein Hmo1p and may regulate its assembly or function	KOG0544

(annotation from the *Saccheromyces* Genome Database)

Filtering with Purification Enrichment scores:

	TFB4	SSL1	TFB1	KIN28	TFB3	CCL1	RAD3	TFB2	SSL2
TFB4		12.8	11.8	1.6	4.6	2.4	0.8	0.8	0
SSL1	12.8		14.1	1.6	3.3	2.4	0	0	0
TFB1	11.8	14.1		0	2.0	0.4	0	0.3	0
KIN28	1.6	1.6	0		3.4	2.8	0	0	0
TFB3	4.6	3.3	2.0	3.4		5.4	1.7	0	0
CCL1	2.4	2.4	0.4	2.8	5.4		0	0	0
RAD3	0.8	0	0	0	1.7	0		0	0
TFB2	0.8	0	0.3	0	0	0	0		0
SSL2	0	0	0	0	0	0	0	0	

In this matrix, a zero in entry (i,j) means that the protein in row i has been co-purified with other proteins, but not with the protein in column j. The order of proteins is the same as in the phylogenetic profile. High similarity in phylogenetic profiles does not correspond to a high propensity to interact (high PE score).

We first remove all components with a zero PE score with all other components, which in this case means we remove SSL2. Subsequently, we cluster the components with single linkage with -1* PE score as distance, we obtain two clusters and remove the smallest cluster. This results in removing TFB2 from the module.

Resulting profile:

KOG	G. lamblia	L. major	P. falciparum	T. parvum	C. parvum	O. tauri	C. merolae	A. thaliana	O. sativa	E. cuniculi	R. oryzae	C. neoformans	U. maydis	S. pombe	Y. lipolytica	C. guillermondii	D. hansenii	C. glabrata	S. cerevisiae	K.lactis	E. gossypii	F. graminearum	N. crassa	S. sclerotiorum	C. immitis	A. nidulans	A. terreus	A. fumigatus	C. elegans	D. melanogaster	H. sapiens	M. musculus	D. discoidum	E. histolytica
KOG2487			1	1	1	1		1	1	1	1	1	1	1	1	1	1	1	1	1	1	1	1	1	1	1	1	1	1	1	1	1	1	1
KOG2807	1	1	1	1	1	1	1	1	1	1	1	1	1	1	1	1	1	1	1	1	1	1	1	1	1	1	1		1	1	2	1	1	2
KOG2074						1		2	1		1	1	1	1	1	1	1	1	1	1	1	1	1	1	1	1	1	1	1	1	1	1	1	
KOG0659		1	1			1	1	3	1	1	1	1	1	1	1	1	1	1	1	1	1	1	1	1	1	1	1	1	2	2	2	4	1	
KOG3800			1	1	1		1	1		1	1	1	1	1	1	1	1	1	1	1	1	1	1	1	1	1	1	1	1	1	1	1	1	
KOG2496						1	1	1			1	1	1	1	1	1	1	1	1	1	1	1	1	1	1	1	1	1	1	1	1	1	10	
KOG1131	1	1	1	1	1	1	2	1	1	2	1	1	1	1	1	1	1	1	1	1	1	1	1	1	1	1	1	1	2	1	1	1	1	2

The resulting submodule has the same raw score (23,0) as the original module, but because it is compared to a different random background than the original module (of smaller random modules, and thus a more dispersed distribution), its cohesiveness score is now 0.979 and the module falls below the cohesiveness threshold.

An other way of filtering with TAP data is to remove those subunits which are more likely to interact with a protein which is not in the module. This results in removing the same proteins as in the other PE filter plus RAD3. There is no change in the raw scores and the cohesiveness score is 0.96.

Trusted KOGs

We compare our KOG-based orthologous groups with ones obtained with the orthoMCL program and trust only those KOGs who have at least 90% overlap with an orthoMCL orthologous group. Based on this criterion, KOG2074, KOG0659, KOG1123 and KOG2496 are regarded as unreliable and removed. This results in a submodule with the same raw scores and a lower cohesiveness score (0.98).

KOG	G. lamblia	L. major	P. falciparum	T. parvum	C. parvum	O. tauri	C. merolae	A. thaliana	O. sativa	E. cuniculi	R. oryzae	C. neoformans	U. maydis	S. pombe	Y. lipolytica	C. guillermondii	D. hansenii	C. glabrata	S. cerevisiae	K.lactis	E. gossypii	F. graminearum	N. crassa	S. sclerotiorum	C. immitis	A. nidulans	A. terreus	A. fumigatus	C. elegans	D. melanogaster	H. sapiens	M. musculus	D. discoidum	E. histolytica
KOG2487			1	1	1	1		1	1	1	1	1	1	1	1	1	1	1	1	1	1	1	1	1	1	1	1	1	1	1	1	1	1	1
KOG2807	1	1	1	1	1	1	1	1	1	1	1	1	1	1	1	1	1	1	1	1	1	1	1	1	1	1	1		1	1	2	1	1	2
KOG2074						1		2	1		1	1	1	1	1	1	1	1	1	1	1	1	1	1	1	1	1	1	1	1	1	1	1	
KOG3800			1	1	1		1	1		1	1	1	1	1	1	1	1	1	1	1	1	1	1	1	1	1	1	1	1	1	1	1	1	
KOG3471			1	1	1		1	1	1	1	1	1	1	1	1	1	1	1	1	1	1	1	1	1	1	1	1	1	1	1	1	1	1	2

Inparalogs

The 50% of orthologous groups with most inparalogs are removed from all modules. This boils down to orthologous groups having more than 7 inparalogs being removed. In our example, this means that KOG2496, KOG0659 and KOG1123 are removed which results in the following profile:

KOG	*G. lamblia*	*L. major*	*P. falciparum*	*T. parvum*	*C. parvum*	*O. tauri*	*C. merolae*	*A. thaliana*	*O. sativa*	*E. cuniculi*	*R. oryzae*	*C. neoformans*	*U. maydis*	*S. pombe*	*Y. lipolytica*	*C. guillermondii*	*D. hansenii*	*C. glabrata*	*S. cerevisiae*	*K.lactis*	*E. gossypii*	*F. graminearum*	*N. crassa*	*S. sclerotiorum*	*C. immitis*	*A. nidulans*	*A. terreus*	*A. fumigatus*	*C. elegans*	*D. melanogaster*	*H. sapiens*	*M. musculus*	*D. discoidum*	*E. histolytica*
KOG2487			1	1	1	1		1	1	1	1	1	1	1	1	1	1	1	1	1	1	1	1	1	1	1	1	1	1	1	1	1	1	1
KOG2807	1	1	1	1	1	1	1	1	1	1	1	1	1	1	1	1	1	1	1	1	1	1	1	1	1	1	1		1	1	2	1	1	2
KOG2074						1		2	1		1	1	1	1	1	1	1	1	1	1	1	1	1	1	1	1	1	1	1	1	1	1	1	
KOG3800			1	1	1		1	1		1	1	1	1	1	1	1	1	1	1	1	1	1	1	1	1	1	1	1	1	1	1	1	1	
KOG1131	1	1	1	1	1	1	2	1	1	2	1	1	1	1	1	1	1	1	1	1	1	1	1	1	1	1	1	1	2	1	1	1	1	2
KOG3471			1	1	1		1	1	1	1	1	1	1	1	1	1	1	1	1	1	1	1	1	1	1	1	1	1	1	1	1	1	1	2

The raw score is unchanged, but the cohesiveness score is now 0.958. Similar results are obtained if one removes orthologous groups based on the fraction of inparalogs instead of the number.

Multifunctional proteins

We have filtered for multifunctional proteins in several ways: one of which was to remove proteins which are also part of an other, nonoverlapping module in the same module definition. The TIIFH complex from MIPS in our example does not contain any multifunctional proteins.

E4: A cohesively evolving pathway: Valine biosynthesis (SGD)

Description:
The valine biosynthesis pathway consists of four steps: in the first step pyruvate resulting from glycolysis is turned into 2-aceto-lactate by ILV2 and ILV6, which in the second step converted to 2,3-dehydroxy-valerate by ILV5, which is then turned into 2-keto-isovalerate by the dehydratase ILV2 and in the final step this 2-keto-isovalerate converted to L-Valine by BAT1 and BAT2.

Subunits:

ORF	gene name	annotation	KOG
YCL009C	ILV6	Regulatory subunit of acetolactate synthase, which catalyzes the first step of branched-chain amino acid biosynthesis; enhances activity of the Ilv2p catalytic subunit, localizes to mitochondria	KOG2663
YLR355C	ILV5	Acetohydroxyacid reductoisomerase, mitochondrial protein involved in branched-chain amino acid biosynthesis, also required for maintenance of wild-type mitochondrial DNA and found in mitochondrial nucleoids	KOG9832
YJR016C	ILV3	Dihydroxyacid dehydratase, catalyzes third step in the common pathway leading to biosynthesis of branched-chain amino acids	KOG2448
YMR108W	ILV2	Acetolactate synthase, catalyses the first common step in isoleucine and valine biosynthesis and is the target of several classes of inhibitors, localizes to the mitochondria; expression of the gene is under general amino acid control	KOG4166
YHR208W	BAT1	Mitochondrial branched-chain amino acid aminotransferase	KOG0975
YJR148W	BAT2	Cytosolic branched-chain amino acid aminotransferase	

(pathway and annotation from the *Saccheromyces* Genome Database)
Subunits BAT1 and BAT2 are the result of the whole genome duplication which occurred in the ancestor of *S. cerevisiae* (Byrne and Wolfe 2005). These subunits are also involved in valine degradation in animals and *L. Major*.

Profile:

	G. lamblia	*L. major*	*P. falciparum*	*T. parvum*	*C. parvum*	*O. tauri*	*C. merolae*	*A. thaliana*	*O. sativa*	*E. cuniculi*	*R. oryzae*	*C. neoformans*	*U. maydis*	*S. pombe*	*Y. lipolytica*	*C. guillermondii*	*D. hansenii*
KOG2663						1	1	2	2		2	1	1	1	1	1	1
KOG9832						1	1	1	2		2	1	1	1	1	1	1
KOG2448						1	1	1	1		2	2	2	1	1	1	1
KOG4166						1	2	1	3		1	1	1	1	1	1	1
KOG0975	1	1				2	2	9	5		4	2	3	1	2	1	1

	C. glabrata	*S. cerevisiae*	*K.lactis*	*E. gossypii*	*F. graminearum*	*N. crassa*	*S. sclerotiorum*	*C. immitis*	*A. nidulans*	*A. terreus*	*A. fumigatus*	*C. elegans*	*D. melanogaster*	*H. sapiens*	*M. musculus*	*D. discoidum*	*E. histolytica*
KOG2663	1	1	1	1	1	1	1	1	1	1	1						
KOG9832	1	1	1	1	1	1	1	1	1	1	1						
KOG2448	1	1	1	1	3	2	2	2	4	6	4						
KOG4166	1	1	1	1	1	1	1	1	1	4	1						
KOG0975	2	2	1	1	6	3	3	2	6	5	4	2	1	2	2	1	

Cohesiveness Scores:

Scores	raw	compared to background
Avg Co-occurrence	0.92	0.995
Avg Deviation From Modular	0.04	0.996
Homogeneous Columns	23	0.99
Species Absent	5	0.97
Species Present	22	0.89
Species Present, Species Absent	(22, 5)	0.99998

Cohesiveness scores for different measures. Species Present, Species Absent is the score used throughout the article. Raw scores are compared to 100000 random modules of the same size, cohesiveness scores for each raw scores are obtained by counting the number of random modules with a better raw score (or in case of a multidimensional method: better raw scores) and dividing that number by 100000.

Filters:

Cross-comparison of module datasets

We keep the combination of the largest number of components which have been found together in a complex or pathway in an other dataset. This module has been confirmed by the 'Pantothenate and CoA biosynthesis', 'Valine, leucine and isoleucine biosynthesis', 'superpathway of isoleucine and valine biosynthesis' and the 'superpathway of leucine, valine, and isoleucine biosynthesis' pathways in the KEGG dataset. Therefore this module passes the cross-comparison filter without losing a subunit.

Filtering with Purification Enrichment scores:

In this filter, we first remove all components with a zero PE score with all other components (in this example ILV3 and ILV2). Subsequently, we cluster the components with single linkage with -1* PE score as distance, we obtain two clusters and remove the smallest cluster. In this case in the last step ILV6 is removed from the module and we are left with a module consisting of one KOG, therefore we can not calculate a score. There are no proteins which are more likely to interact with a protein outside the module than with a fellow module member.

	ILV6	ILV5	ILV3	ILV2	BAT1	BAT2
ILV6		*0.5*	0	0	0	0
ILV5	*0.5*		0	0	0	0
ILV3	0	0		0	0	0
ILV2	0	0	0		0	0
BAT1	0	0	0	0		5
BAT2	0	0	0	0	5	

Trusted KOGs

We compare our KOG-based orthologous groups with ones obtained with the orthoMCL program and trust only those KOGs who have at least 90% overlap with an orthoMCL orthologous group. Based on this criterion, KOG2448, KOG4166 and KOG0975 are regarded as unreliable and removed. This results in a submodule with a better raw score (22, 12) but a lower cohesiveness score (0.99986). This perfectly cohesive pattern is not considered cohesive, because, due to the underlying phylogeny, multiple orthologous groups show this pattern of presence and absence and have the same raw score.

	G. lamblia	*L. major*	*P. falciparum*	*T. parvum*	*C. parvum*	*O. tauri*	*C. merolae*	*A. thaliana*	*O. sativa*	*E. cuniculi*	*R. oryzae*	*C. neoformans*	*U. maydis*	*S. pombe*	*Y. lipolytica*	*C. guillermondii*	*D. hansenii*	*C. glabrata*	*S. cerevisiae*	*K.lactis*	*E. gossypii*	*F. graminearum*	*N. crassa*	*S. sclerotiorum*	*C. immitis*	*A. nidulans*	*A. terreus*	*A. fumigatus*	*C. elegans*	*D. melanogaster*	*H. sapiens*	*M. musculus*	*D. discoidum*	*E. histolytica*
KOG2663						1	1	2	2		2	1	1	1	1	1	1	1	1	1	1	1	1	1	1	1	1	1						
KOG9832						1	1	1	2		2	1	1	1	1	1	1	1	1	1	1	1	1	1	1	1	1	1						

Inparalogs

When we remove orthologous groups which are part of the 50% of orthologous groups with most inparalogs, we remove the only untrusted orthologous groups. The result is the same as described in the paragraph above.

E5: A flexibly evolving pathway: Valine degradation (SGD)

Description:

Branched chain and aromatic amino acids are degraded by *S. cerevisiae* via the Ehrlich pathway, which consists of the following three steps: deamination of the amino acid, decarboxylation of the resulting alpha-keto acid and the reduction of the resulting aldehyde to alcohol.

Subunits:

ORF	gene name	annotation	KOG
YDL168W	SFA1	Bifunctional enzyme containing both alcohol dehydrogenase and glutathione-dependent formaldehyde dehydrogenase activities, functions in formaldehyde detoxification and formation of long chain and complex alcohols, regulated by Hog1p-Sko1p	KOG0022
YGL256W	ADH4	Alcohol dehydrogenase isoenzyme type IV, dimeric enzyme demonstrated to be zinc-dependent despite sequence similarity to iron-activated alcohol dehydrogenases; transcription is induced in response to zinc deficiency	KOG3857
YHR208W	BAT1	Mitochondrial branched-chain amino acid aminotransferase	KOG0975
YJR148W	BAT2	Cytosolic branched-chain amino acid aminotransferase	
YLR134W	PDC5	Minor isoform of pyruvate decarboxylase, key enzyme in alcoholic fermentation, decarboxylates pyruvate to acetaldehyde, regulation is glucose- and ethanol-dependent, repressed by thiamine, involved in amino acid catabolism	KOG1184
YGR087C	PDC6	Minor isoform of pyruvate decarboxylase, decarboxylates pyruvate to acetaldehyde, involved in amino acid catabolism; transcription is glucose- and ethanol-dependent, and is strongly induced during sulfur limitation	
YLR044C	PDC1	Major of three pyruvate decarboxylase isozymes, key enzyme in alcoholic fermentation, decarboxylates pyruvate to acetaldehyde; subject to glucose-, ethanol-, and autoregulation; involved in amino acid catabolism	
YBR145W	ADH5	Alcohol dehydrogenase isoenzyme V; involved in ethanol production	KOG0023
YMR083W	ADH3	Mitochondrial alcohol dehydrogenase isozyme III; involved in the shuttling of mitochondrial NADH to the cytosol under anaerobic conditions and ethanol production	
YMR303C	ADH2	Glucose-repressible alcohol dehydrogenase II, catalyzes the conversion of ethanol to acetaldehyde; involved in the production of certain carboxylate esters; regulated by ADR1	
YOL086C	ADH1	Alcohol dehydrogenase, fermentative isozyme active as homo- or heterotetramers; required for the reduction of acetaldehyde to ethanol, the last step in the glycolytic pathway	

(pathway and annotation from the *Saccheromyces* Genome Database)

Subunits BAT1 and BAT2 are ohnologs. The same goes for PDC1, PDC5 and PDC6. Unlike ADH1, ADH2, ADH3 and ADH5, ADH4 contains the PFAM domain 'Fe-ADH' associated with the orthologous group KOG3857'. Some fungal members of this orthologous group have a duplicate which, apart from the 'Fe-ADH' domain, contains an additional 'DHQ_synthase'. (Finn et al. 2008)

Profile:

KOG	*G. lamblia*	*L. major*	*P. falciparum*	*T. parvum*	*C. parvum*	*O. tauri*	*C. merolae*	*A. thaliana*	*O. sativa*	*E. cuniculi*	*R. oryzae*	*C. neoformans*	*U. maydis*	*S. pombe*	*Y. lipolytica*	*C. guillermondii*	*D. hansenii*
KOG0022						1	1	10	12			1	3	2	1	1	1
KOG3857	4	1			1	1					2	1	2	1	1		
KOG1184		1			1			4	5		3	1	1	4	2	3	4
KOG0023		1			1	3	1	9	12		12	7	6	1	5	4	6
KOG0975	1	1				2	2	9	5		4	2	3	1	2	1	1

KOG	*C. glabrata*	*S. cerevisiae*	*K.lactis*	*E. gossypii*	*F. graminearum*	*N. crassa*	*S. sclerotiorum*	*C. immitis*	*A. nidulans*	*A. terreus*	*A. fumigatus*	*C. elegans*	*D. melanogaster*	*H. sapiens*	*M. musculus*	*D. discoidum*	*E. histolytica*
KOG0022	1	1	1	1	2	2	2	2	5	5	2	2	1	7	6	1	
KOG3857		1	1		3	1	2	2	3	2	3	1	1	1	1	1	11
KOG1184	4	5	3	4	3	3	1	2	2	5	3						
KOG0023	4	6	5	3	16	6	10	6	13	14	10	3				8	
KOG0975	2	2	1	1	6	3	3	2	6	5	4	2	1	2	2	1	

Cohesiveness Scores:

Scores	raw	compared to background
Avg Co-occurrence	0.75	0.71
Avg Deviation From Modular	0.14	0.75
Homogeneous Columns	16	0.67
Species Absent	3	0.9
Species Present	13	0.6
Species Present, Species Absent	(13, 3)	0.98

Cohesiveness scores for different measures. Species Present, Species Absent is the score used throughout the article. Raw scores are compared to 100000 random modules of the same size, cohesiveness scores for each raw scores are obtained by counting the number of random modules with a better raw score (or in case of a multidimensional method: better raw scores) and dividing that number by 100000.

Filters:

Cross-comparison of module datasets

We keep the combination of the largest number of components which have been found together in a complex or pathway in an other dataset. Only part of this module (proteins ADH5, SFA1, ADH4, PDC6, PDC1, PDC5, ADH3, ADH2 and ADH1) overlaps with the KEGG Glycolysis/Gluconeogenesis pathway. The proteins removed are all duplicates, so the set of KOGs and the scores remain the same.

KOG	*G. lamblia*	*L. major*	*P. falciparum*	*T. parvum*	*C. parvum*	*O. tauri*	*C. merolae*	*A. thaliana*	*O. sativa*	*E. cuniculi*	*R. oryzae*	*C. neoformans*	*U. maydis*	*S. pombe*	*Y. lipolytica*	*C. guillermondii*	*D. hansenii*	*C. glabrata*	*S. cerevisiae*	*K.lactis*	*E. gossypii*	*F. graminearum*	*N. crassa*	*S. sclerotiorum*	*C. immitis*	*A. nidulans*	*A. terreus*	*A. fumigatus*	*C. elegans*	*D. melanogaster*	*H. sapiens*	*M. musculus*	*D. discoideum*	*E. histolytica*
KOG0022						1	1	10	12			1	3	2	1	1	1	1	1	1	1	2	2	2	2	5	5	2	2	1	7	6	1	
KOG3857	4	1			1	1					2	1	2	1	1				1	1		3	1	2	2	3	2	3	1	1	1	1	1	11
KOG1184		1			1			1	1	1	1	1	1	4	1	1	1	4	4	5	3	4	3	3	2	2	5	3	1					
KOG0023		1			1	3	1	9	12		12	7	6	1	5	4	6	4	6	5	3	16	6	10	6	13	14	10	3				8	

Filtering with Purification Enrichment scores:

	SFA1	ADH4	BAT1	BAT2	PDC5	PDC6	PDC1	ADH5	ADH3	ADH2	ADH1
SFA1		0	0	0	0	0	0	0	0	0	0
ADH4	0		*0.7*	0	0	0	0	0	0	0	0
BAT1	0	*0.7*		5.0	0	0	0	0	0	0	0
BAT2	0	0	5.0		0	0	0	0	0	0	0
PDC5	0	0	0	0		0	2.0	0	*0.6*	0	0
PDC6	0	0	0	0	0		*1.3*	2.7	*0.9*	1.7	0
PDC1	0	0	0	0	2.0	*1.3*		*1.5*	2.2	4.0	0
ADH5	0	0	0	0	0	2.7	*1.5*		2.5	3.6	0
ADH3	0	0	0	0	*0.6*	*0.9*	2.2	2.5		5.3	0
ADH2	0	0	0	0	0	1.7	4.0	3.6	5.3		0
ADH1	0	0	0	0	0	0	0	0	0	0	

In this filter, we first remove all components with a zero PE score with all other components and cluster the remaining components with single linkage with -1* PE score as distance, we obtain two clusters and remove the smallest cluster. We apply this filter only to those modules of which all members have at least one interaction with an associated confidence score >= 0.2 and this pathway has some members which do not interact with any other protein. So we don't apply the filter here. The matrix with PE scores between module members id printed below.

Trusted KOGs

We compare our KOG-based orthologous groups with ones obtained with the orthoMCL program and trust only those KOGs who have at least 90% overlap with an orthoMCL orthologous group. Members of this module are all assigned to untrusted orthologous groups, therefore this filter removes the entire module.

Inparalogs

We remove orthologous groups which are part of the 50% of orthologous groups with most inparalogs. All orthologous groups associated with this module belong to this top 50% and therefore the filter removes the entire module.

Multifunctional proteins

Protein SFA1 is also involved in other pathways in the GSD dataset: the glutathione dependent formaldehyde oxidation II pathway and leucine, isoleucine, phenylalanine and tryptophan degradation. Removal of this protein does improve the raw score ((15,3) instead of (13,3)) and the cohesiveness score relative to the random background is higher: 0.97 instead of 0.9.

References

Byrne K.P., Wolfe K.H. 2005. The Yeast Gene Order Browser: combining curated homology and syntenic context reveals gene fate in polyploid species. *Genome Res.* **15**: 1456-61

Finn R.D., Tate J., Mistry J., Coggill P.C., Sammut J.S., Hotz H.R., Ceric G., Forslund K., Eddy S.R., Sonnhammer E.L. et al. 2008. The Pfam proteins database. *Nucleic Acids Research* **36**:D281-D288

Gavin AC., Aloy P., Grandi P., Krause R., Boesche M., Marzioch M., Rau C., Jensen L.J., Bastuck S., Dümpelfeld B. et al. 2006. Proteome survey reveals modularity of the yeast cell machinery. *Nature* **440**: 631-636.

Grandi P., Schlaich N., Tekotte H., Hurt E.C.. 1995. Functional interaction of Nic96p with a core nucleoporin complex consisting of Nsp1p, Nup49p and a novel protein Nup57p. *EMBO J.* **14**:76-87

Grandi P., Emig S., Weise C., Hucho F., Pohl T., Hurt E.C. 1995. A novel nuclear pore protein Nup82p which specifically binds to a fraction of Nsp1p. *J. Cell. Biol.* **130**:1263-73

Saccheromyces Genome Database ftp://ftp.yeastgenome.org/yeast/ June 2008

Schrader N., Stelter P., Flemming D., Kunze R, Hurt E., Vetter I.R. 2008. Structural basis of the nic96 subcomplex organization in the nuclear pore channel. 2008. *Mol. Cell.* **29**:46-55

Yao W., Lutzmann M., Hurt E. 2008. A versatile interaction platform on the Mex67-Mtr2 receptor creates an overlap between mRNA and ribosome export. *EMBO J.* **27**:6-16
